# Tumour‐derived SAA1 reprogrammes macrophages to promote CXCL1‐mediated metastasis in Ovarian Cancer

**DOI:** 10.1002/ctm2.70698

**Published:** 2026-05-18

**Authors:** Xuan Zhou, Huangyang Meng, Qianjing Chang, Jingjing Ren, Meichen Wen, Lin Zhang, Wenjun Cheng

**Affiliations:** ^1^ Department of Gynecology The First Affiliated Hospital of Nanjing Medical University Nanjing Jiangsu China

**Keywords:** CXCL1, macrophage, metastasis, ovarian cancer, SAA1, tumour microenvironment

## Abstract

Patients with advanced high‐grade serous ovarian carcinoma (HGSOC) often experience extremely poor prognoses due to extensive peritoneal metastases, yet the underlying driving mechanisms remain unclear. This study reveals that serum amyloid A1 (SAA1) is markedly upregulated in late‐stage HGSOC and is closely associated with poor prognosis and distant metastasis. These findings were validated through both single‐cell RNA sequencing and clinical specimen analyses. Surprisingly, SAA1 does not directly promote tumour cell proliferation or migration. Instead, it reshapes the immunosuppressive tumour microenvironment by activating FPR2^+^ tumour‐associated macrophages (TAMs). Mechanistically, the SAA1–FPR2 signalling axis triggers the JAK2/STAT3 pathway in macrophages, enhancing the transcription and secretion of CXCL1. This, in turn, induces epithelial–mesenchymal transition (EMT) in tumour cells and endows them with greater metastatic potential. Animal models further confirmed that SAA1 knockdown, macrophage depletion, or CXCL1 blockade all significantly suppressed peritoneal dissemination. Collectively, this study identifies a novel SAA1–TAM–CXCL1 immune‐inflammatory signalling axis, elucidates its critical role in ovarian cancer metastasis, and provides a robust theoretical foundation for the development of innovative anti‐metastatic therapeutic strategies.

**Key points:**

- A distinct SAA1‐enriched tumour cell subset with metastasis‐associated features is identified in ovarian cancer.
- Tumour‐derived SAA1 reprograms TAM through FPR2‐mediated JAK2–STAT3 signalling to induce an immunosuppressive phenotype.
- The SAA1–TAM–CXCL1 axis facilitates metastatic progression in ovarian cancer.

## Graphical abstract

1. A distinct SAA1‐enriched tumour cell subset with metastasis‐associated features is identified in ovarian cancer.
2. Tumour‐derived SAA1 reprograms TAM through FPR2‐mediated JAK2–STAT3 signalling to induce an immunosuppressive phenotype.
3. The SAA1–TAM–CXCL1 axis facilitates metastatic progression in ovarian cancer.

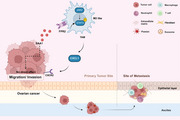

## 1 INTRODUCTION

High‐grade serous ovarian carcinoma (HGSOC) represents the most lethal subtype among gynaecologic malignancies. Approximately 70% of patients are diagnosed at an advanced stage, with a 5‐year survival rate of less than 40%.^1^, ^2^ The most prominent characteristic of HGSOC is extensive peritoneal dissemination, occurring in more than 70% of cases, often accompanied by diffuse multifocal metastases and malignant ascites.^3^, ^4^, ^5^ Such peritoneal spread not only increases the complexity of cytoreductive surgery but is also closely associated with chemotherapy resistance and a high recurrence rate, constituting a key factor in poor patient prognosis.^6^, ^7^ Although surgery combined with chemotherapy can induce initial remission, overall survival has not significantly improved. This indicates that the current therapeutic strategies remain insufficient to fundamentally alter the natural course of disease metastasis. Therefore, elucidating the key molecular and cellular interaction mechanisms that drive peritoneal dissemination in ovarian cancer is of critical importance for improving patient prognosis and developing novel targeted therapeutic strategies.

To address the challenging problem of ovarian cancer metastasis, numerous studies have explored its underlying mechanisms. For instance, Cui et al. discovered that targeting tumour‐derived exosomal miRNAs can suppress ovarian cancer invasion and migration.^8^ Nuzhat Ahmed et al. found that Oct4A endows ovarian cancer cells with stem cell–like properties and metastatic potential.^9^ However, most of these studies have relied on bulk tumour analyses, overlooking the high cellular heterogeneity of ovarian cancer. In fact, distinct epithelial subpopulations display substantial differences in their invasive capacity, immune evasion potential and colonization ability.^10^ Identifying the key cellular subsets that possess genuine metastatic potential is therefore essential for elucidating the mechanisms underlying disease progression and for defining precise therapeutic targets.

Notably, the completion of metastasis is not solely determined by the intrinsic potential of tumour cells but also critically depends on the tumour microenvironment (TME).^11^ TAMs are among the most abundant immune cell populations within the ovarian cancer tumour microenvironment and exhibit remarkable functional plasticity, displaying either pro‐inflammatory or immunosuppressive characteristics depending on the specific signalling stimuli they encounter.^12^, ^13^ However, most current studies still focus on TAM polarization and their roles in immunosuppression or metastasis promotion, with limited understanding of how tumour cell heterogeneity actively shapes TAM phenotypes.^14^, ^15^ Previous research has suggested that certain tumour‐secreted factors (such as IL‐6, CSF1 and CCL2) can induce TAMs to acquire a tumour‐promoting phenotype.^16^, ^17^ Yet, in ovarian cancer, a highly heterogeneous disease, it remains unclear whether specific epithelial subpopulations selectively educate TAMs through the secretion of key factors to drive their transition toward a pro‐metastatic state. Elucidating this intercellular communication process could not only clarify the core mechanisms underlying peritoneal dissemination but also provide new insights for immunotherapy and targeted intervention.

In this study, we performed single‐cell transcriptomic profiling of matched primary and metastatic lesions from HGSOC patients and identified a critical epithelial subpopulation characterized by high SAA1 expression. Further molecular and functional validation revealed that SAA1 deficiency did not significantly affect ovarian cancer cell growth or migration. Instead, SAA1 activated the FPR2 signalling pathway in macrophages, inducing their polarization toward an immunosuppressive macrophage phenotype and upregulating CXCL1 transcription via the JAK2/STAT3 axis. The secreted CXCL1 subsequently promoted EMT and enhanced tumour cell invasiveness, thereby accelerating ovarian cancer metastasis. Our findings uncover a pivotal role for SAA1‐mediated macrophage reprogramming in ovarian cancer metastasis and propose a novel therapeutic strategy aimed at remodelling the immune microenvironment and inhibiting tumour dissemination by targeting the SAA1–TAM–CXCL1 signalling circuit.

## 2 MATERIALS AND METHODS

### 2.1 Animal experiment

All animal experiments were approved by the Animal Ethics Committee of the First Affiliated Hospital of Nanjing Medical University (IACUC‐2505045). Female C57BL/6 mice (6–8 weeks old) were randomly assigned to experimental groups (*n* = 5 per group) and intraperitoneally injected with 5 × 10^6^ luciferase‐labelled ID8 ovarian cancer cells with stable SAA1 knockdown (shSAA1‐ID8‐LUC) or control cells. Mice were divided into six groups: shCtrl, shSAA1, shCtrl + control liposomes, shCtrl + clodronate liposomes, shCtrl + DMSO, and shCtrl + SB225002. Macrophage depletion was achieved by intraperitoneal administration of clodronate liposomes, and CXCR2 inhibition was performed using SB225002, with corresponding vehicles as controls. Tumour burden was monitored by in vivo bioluminescence imaging. At the experimental endpoint, mice were euthanized, and peritoneal tumours and major organs were collected for analysis.

### 2.2 Cell culture and reagents

SKOV3, OVCAR3, THP‐1 and ID8 cell lines were obtained from ATCC and authenticated by STR profiling. ID8 cells were cultured in DMEM, and SKOV3, OVCAR3, and THP‐1 cells in RPMI‐1640, each supplemented with 10% FBS and 1% penicillin–streptomycin, at 37°C with 5% CO_2_. Recombinant human SAA1 (ABclonal) and CXCL1 (Novoprotein, C597) were reconstituted in PBS containing 0.1% BSA. WRW4 and STATTIC (MedChemExpress, HY‐13818) were used as indicated.

### 2.3 Cell immunofluorescence

Cells were seeded in confocal dishes (1 × 10^4^ cells/well) and cultured for 24 h. After fixation, permeabilization and blocking, cells were incubated with primary antibodies at 4°C overnight, followed by fluorescent secondary antibodies for 2 h. Nuclei were stained with DAPI, and images were acquired using a Zeiss LSM 880 confocal microscope with a 63× oil‐immersion objective. Antibodies are listed in Table S1.

### 2.4 Cellular thermal shift assay (CETSA)

CETSA was performed as described previously with minor modifications.^18^ Cells were treated with WRW4 (50 µM) or DMSO for 24 h, harvested, and resuspended in PBS with protease inhibitors. Aliquots were heated at 37°C–63°C for 3 min, rapidly cooled, subjected to two freeze–thaw cycles, centrifuged (20 000 × g, 20 min, 4°C), and the supernatants were analysed by Western blot.

### 2.5 Cell transfections

Lentiviral particles and polybrene were purchased from Vigene Biosciences and used for cell transduction according to the manufacturer's instructions.

### 2.6 Cell viability assay and colony formation assay

Cell viability was measured using a CCK‐8 kit (Beyotime, C0038) according to the manufacturer's instructions. For colony formation, cells were seeded in 6‐well plates at a density of 1000 cells per well and cultured as indicated for 14 days. Colonies were fixed with 4% paraformaldehyde (Biosharp, BL539A) and stained with crystal violet (Beyotime, C0121).

### 2.7 CRISPR/Cas9 knockout

sgRNAs to early coding exons were designed online with off‐target minimization. Two candidate sequences (LM2401024A: GCUGCCAAAAGGGGACCUGG; LM2401024B: CGUGAUCACUUCUGCAGCCC) were selected. Ribonucleoprotein (RNP) complexes were assembled by incubating Cas9 protein with sgRNAs at a 1:8 molar ratio and delivered into cells by electroporation. Following recovery in complete medium, single‐cell sorting was performed at 72 hours using the DispenCell platform. Expanded clones were genotyped by PCR and confirmed by Sanger sequencing (TsingKe). Matched Cas9WT control clones were generated in parallel for comparison. Knockout clones with stable growth were cryopreserved for subsequent experiments.

### 2.8 Chromatin immunoprecipitation (ChIP)

ChIP assays were performed using the Pierce ChIP Kit (Thermo Fisher Scientific, 26156). Macrophages were crosslinked with 1% formaldehyde, lysed, and digested with MNase. Chromatin was immunoprecipitated with normal IgG or anti‐STAT3 antibody (Proteintech, 10253‐2‐AP) at 4°C overnight, followed by Protein A/G bead enrichment. After reversal of crosslinks, DNA was purified and subjected to PCR analysis. Primer sequences are listed in Table S2.

### 2.9 Dual‐luciferase reporter assay

To assess the transcriptional regulation of CXCL1 by STAT3, the wild‐type or mutated CXCL1 promoter fragments were inserted into the psiCHECK2 vector and introduced into cells together with STAT3 expression plasmids using Lipofectamine 3000 (Thermo Fisher Scientific, L3000). After 48 h, luciferase activities were quantified with the Dual‐Luciferase Reporter Assay System (Promega).

### 2.10 ELISA detection

CXCL1 levels in macrophage supernatants were measured using a human CXCL1 ELISA kit (Huabio, EH0054) following the manufacturer's instructions.

### 2.11 Flow cytometry analysis

Macrophages were prepared as single‐cell suspensions and stained with PE‐Cy7–conjugated anti‐human CD86 (BioLegend, 305422), APC–conjugated anti‐human CD206 (BioLegend, 321110), and 7‐AAD (BioLegend, 420404) for 1 h at 4°C. After two washes with flow cytometry buffer, cells were resuspended in 0.5 mL buffer and analysed using a FACSCalibur flow cytometer (BD Biosciences). Data were processed with FlowJo software (Tree Star).

### 2.12 Immunohistochemistry (IHC) and tissue immunofluorescence (IF)

Paraffin‐embedded ovarian cancer tissue sections were processed for IHC by incubating with primary antibodies overnight at 4°C, followed by HRP‐conjugated secondary antibodies and chromogenic detection. For tissue immunofluorescence, staining was performed using the tyramide signal amplification (TSA) method with DAPI nuclear counterstaining. Images were acquired using a Thunder Imaging System (Leica). Antibodies are listed in Table S1. Image quantification for IF and IHC was performed using ImageJ. Unless otherwise specified, three randomly selected non‐overlapping fields were analysed for each sample under the same magnification. Images from the same staining batch were quantified using a uniform threshold and identical measurement settings to reduce batch‐related variability. Positive staining signals were quantified using a threshold‐based approach, and the mean value from the three fields was used as one biological data point for statistical analysis.

### 2.13 Macrophage treatment

THP‐1 monocytes were differentiated into M0‐like macrophages with 200 nM PMA (MedChemExpress, HY‐18739) for 24 h, followed by a 24 h rest. To generate tumour‐educated macrophage‐conditioned medium (TeMCM), macrophages were exposed to conditioned medium from SKOV3^Cas9^WT or SKOV3^SAA1–/–^ cells for 24 h, washed with PBS, and cultured in fresh complete medium for an additional 24 h. The supernatant was collected for downstream assays on epithelial cells or tumour organoids. Depending on the experimental group, macrophages were treated with WRW4 (10 µM; MedChemExpress, HY‐P1119) or recombinant human SAA1 (1 µg/mL; ABclonal, RP01095) for 24 h, with vehicle as control. This procedure ensures that TeMCM reflects macrophage‐derived secretions induced by tumour education rather than residual tumour medium.

### 2.14 Patient samples and establishment of PDOs

Patient‐derived organoids (PDOs) and the corresponding parental tumours were obtained from newly diagnosed ovarian cancer patients at the First Affiliated Hospital of Nanjing Medical University with institutional ethics approval. PDOs were established from fresh tumour tissues following the protocol of Kopper et al.^19^ Briefly, specimens were cleared of blood and necrotic debris, minced and digested with collagenase and dispase at 37°C. The resulting multicellular clusters were passed through a 70 µm strainer, embedded in matrix gel (BME), and seeded into 48‐well plates. After gel solidification for 20 min at 37°C, 200 µL organoid growth medium was added per well. Medium was replaced every 3 days, and organoids were passaged every 2–3 weeks. Five PDO lines were used in the functional assays. The composition of the organoid growth medium is provided in Table S3.

### 2.15 Transwell migration and invasion

Cell migration and invasion were assessed using 24‐well Transwell chambers with 8‐µm pores (Corning, 3422); invasion assays included Matrigel coating (Corning, 354234). Cells (4 × 10^4^) were seeded in the upper chambers, with 10% FBS in the lower chambers as chemoattractant. After 24–48 h, migrated or invaded cells were fixed, crystal violet–stained (Beyotime, C0121), and quantified using ImageJ. For co‐culture assays, tumour cells were seeded in the upper chambers and macrophages in the lower chambers.

### 2.16 Western blot

Total proteins were extracted with RIPA buffer (Beyotime, P0013B), quantified by a BCA kit (Beyotime, P0012), separated by SDS‐PAGE, and transferred to PVDF membranes (Millipore, IPVH00010). Membranes were incubated with primary and HRP‐conjugated secondary antibodies, and signals were visualized using an ECL kit (Biosharp, BL523A). Antibodies used for Western blotting are listed in Table S1.

### 2.17 Single‐cell analysis of ovarian cancer

Fresh primary and peritoneal metastatic HGSOC tumours were collected from the First Affiliated Hospital of Nanjing Medical University. Single‐cell suspensions were prepared and processed using the 10x Genomics Chromium platform, followed by paired‐end sequencing on an Illumina NovaSeq 6000. Raw data were processed with Cell Ranger and analysed using Seurat, with batch correction performed by Harmony. Cell clustering, marker‐based cell annotation, differential expression analysis between primary and metastatic epithelial cells and cell–cell communication analysis were performed using Seurat and CellChat, with a focus on SAA1/SAA2‐mediated epithelial–TAM signalling. For trajectory inference, epithelial cells were further analysed using Monocle 2 with highly variable genes as ordering genes. Dimensionality reduction and trajectory reconstruction were performed using DDRTree, followed by pseudotime ordering. For external validation, public 10x Genomics scRNA‐seq data were obtained from GEO under accession number GSE222556. Four untreated ovarian cancer patients with paired primary and metastatic lesions were selected for reanalysis, comprising eight samples: GSM6925966, GSM6925968, GSM6925972, GSM6925973, GSM6925976, GSM6925978, GSM6925993 and GSM6925995. Seurat‐based quality control was performed to remove low‐quality cells, potential doublets, and cells with high mitochondrial gene content before downstream analysis.

### 2.18 Statistical analysis

Data are expressed as mean ± SD. Statistical tests were performed with GraphPad Prism 10.0. Two‐group comparisons used unpaired Student's *t*‐test, and multiple groups used one‐way ANOVA. Survival analysis applied Kaplan–Meier with log‐rank test. Statistical significance: ns, not significant; *p < 0.05; **p < 0.01; ***p < 0.001; ****p < 0.0001.

## 3 RESULTS

### 3.1 Single‐cell RNA sequencing reveals SAA1–associated metastatic epithelial subpopulations in ovarian cancer

**FIGURE 1:**
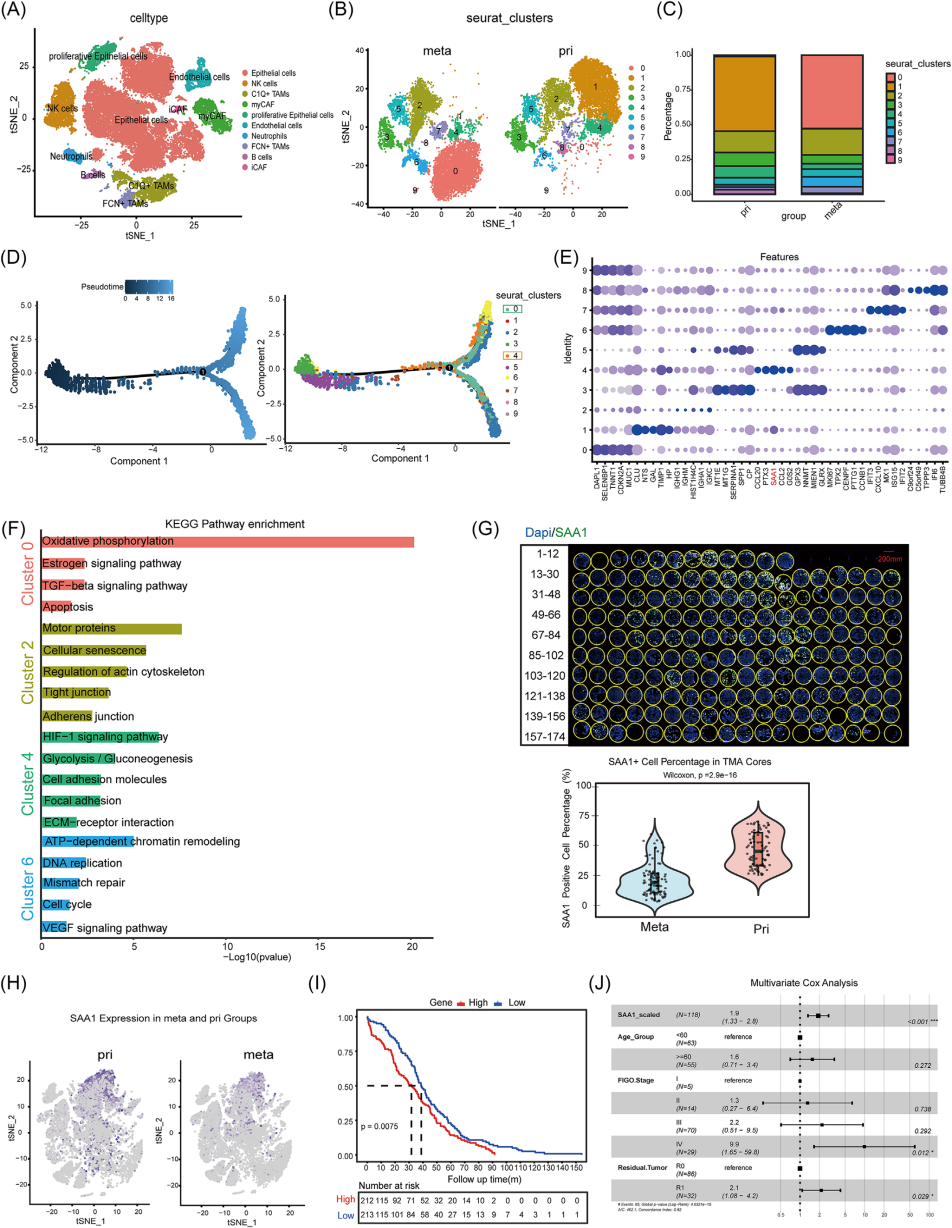
Identification of SAA1‐associated metastatic epithelial subpopulations in ovarian cancer by scRNA‐seq. (A) t‐SNE visualization of cells from paired primary tumours and peritoneal metastases of two patients. (B) Distribution of the ten epithelial clusters in Pri and Meta samples, shown separately. (C) Relative proportions of the ten epithelial clusters in Pri and Meta samples. (D) Pseudotime analysis of epithelial cells from metastases. Left: cells ordered along the inferred trajectory from early (dark) to late (light); the black line indicates the main lineage with bifurcation. Right: same embedding coloured by clusters (0–9). (E) Dot plot showing representative gene expression across the ten clusters; dot size indicates the fraction of expressing cells, and colour denotes average expression. (F) KEGG enrichment of clusters 0, 2, 4 and 6 based on cluster‐specific marker genes. (G) Representative TMA cores stained for SAA1 (green) and nuclei (blue). Scale bar, 200 µm. Quantification of SAA1^+^ cell percentage in Pri and Meta TMA cores is shown as violin plots with overlaid box plots (Pri, *n* = 81; Meta, *n* = 93). *p* value was determined by the Wilcoxon rank‐sum test, *p* = 2.9 × 10^−^^1^ ^6^. (H) t‐SNE plots showing SAA1 expression in primary (pri) and metastatic (meta) ovarian cancer cells. (I) Kaplan–Meier survival curves in the TCGA‐OV cohort (*n* = 429) according to SAA1 expression (log‐rank *p* = 0.0075). (J) Multivariate Cox regression forest plot identifying independent prognostic factors for ovarian cancer, including SAA1 expression and clinicopathological variables.

To minimize inter‐patient variability, we selected two late‐stage HGSOC patients with comparable clinical characteristics, obtaining a total of four matched samples for single‐cell RNA sequencing. After stringent quality control and removal of doublets and mitochondrial gene–enriched cells, 35 024 high‐quality cells were retained for downstream analyses. Unsupervised clustering based on t‐SNE identified ten major cell populations, which were annotated using canonical markers: epithelial cells (EPCAM, KRT8, MUC16), NK cells (NKG7), C1Q^+^ TAMs (C1QA, APOE), fibroblast‐like CAFs (DCN, COL3A1, ACTA2), proliferating epithelial cells (TOP2A, MKI67), endothelial cells (VWF, PLVAP, KDR), neutrophils (FCGR3B), FCN1^+^ TAMs (FCN1, CD86), B cells (CD79A) and inflammatory CAFs (CXCL12, PDGFRA) (Figures 1A and S1A). Comparison between primary and metastatic lesions revealed that although both were predominantly composed of epithelial cells, the metastatic sites exhibited a markedly higher proportion of immune and stromal cells, consistent with the features of the peritoneal microenvironment. This finding suggests significant compositional differences between tumour sites (Figure S1B).

In light of the well‐known heterogeneity of ovarian cancer cells, we performed a refined subclustering analysis of the epithelial compartment. Based on highly variable genes, ten epithelial subclusters (0–9) were identified, each displaying distinct marker gene expression patterns (Figures 1E and S1C), further confirming the extensive cellular heterogeneity within the epithelial population. Analysis by tumour origin revealed notable distributional differences between primary and metastatic epithelial cells (Figure 1B). Considering that ovarian cancer dissemination follows a sequential process—detachment from the primary site, transcoelomic migration, implantation, and expansion at secondary sites—we hypothesized that certain epithelial subpopulations may be shared between primary and metastatic tumours and could represent early migratory initiator cells.

Trajectory inference analysis revealed that within metastatic epithelial cells, clusters 0 and 4 formed critical branching points, evolving respectively towards clusters 2 and 6 (Figure 1D). Of note, cluster 0 was almost exclusively derived from metastatic lesions, whereas cluster 4 could already be detected in primary tumours (Figure 1C). Functional enrichment analysis of the epithelial subclusters further delineated their biological characteristics: cluster 0 was enriched in oxidative phosphorylation, oestrogen response, and TGF‐β signalling pathways; cluster 6 was associated with DNA replication, cell cycle progression, VEGF and HIF‐1 signalling, indicating a proliferative phenotype; cluster 2 was related to cytoskeleton regulation, senescence, and adherend junctions; while cluster 4 showed prominent enrichment in glycolysis, cell adhesion, ECM–receptor interaction and chemotaxis pathways, implying enhanced matrix sensing and migratory potential (Figure 1F). In contrast, clusters 1, 5 and 7 were predominantly associated with viral/bacterial infection‐related pathways and immune responses, whereas cluster 3 was mainly linked to protein processing, extracellular matrix organization and cellular structural regulation. Notably, cluster 8 showed a strong enrichment in pathways related to neurodegenerative diseases (Figure S1D).

Consistent with its early pseudotime trajectory and enrichment in migration‐related pathways, cluster 4 displayed features characteristic of metastasis‐initiating cells. Remarkably, SAA1 emerged as a defining marker gene of cluster 4, with expression patterns consistent with its transcriptional state and distribution, suggesting that SAA1^+^ epithelial cells may represent early disseminating populations (Figure 1E). Single‐cell analysis showed SAA1 enrichment in a tumour‐cell subset and a primary‐biased pattern compared with metastases (Figure 1H). To further validate this observation, we re‐analysed an independent public single‐cell RNA‐seq dataset of ovarian cancer. Major cell populations were clearly identified in this cohort (Figure S1E), and SAA1 expression was again detected in epithelial cells with a primary‐biased distribution compared with metastatic lesions (Figure S1F–H). Consistently, immunofluorescence staining of matched ovarian cancer samples confirmed significantly higher SAA1 expression in primary lesions than in metastatic sites (Figure 1G), supporting a potential role of SAA1^+^ tumour cells in metastasis initiation. Analysis of the TCGA‐OV cohort showed that high SAA1 expression was significantly associated with shorter overall survival (Figure 1I). In our clinical data, SAA1 expression remained associated with poor overall survival in multivariate Cox regression analysis after adjustment for clinical covariates (Figure 1J).

### 3.2 Tumour‐derived SAA1 promotes ovarian cancer cell metastasis through macrophages

**FIGURE 2:**
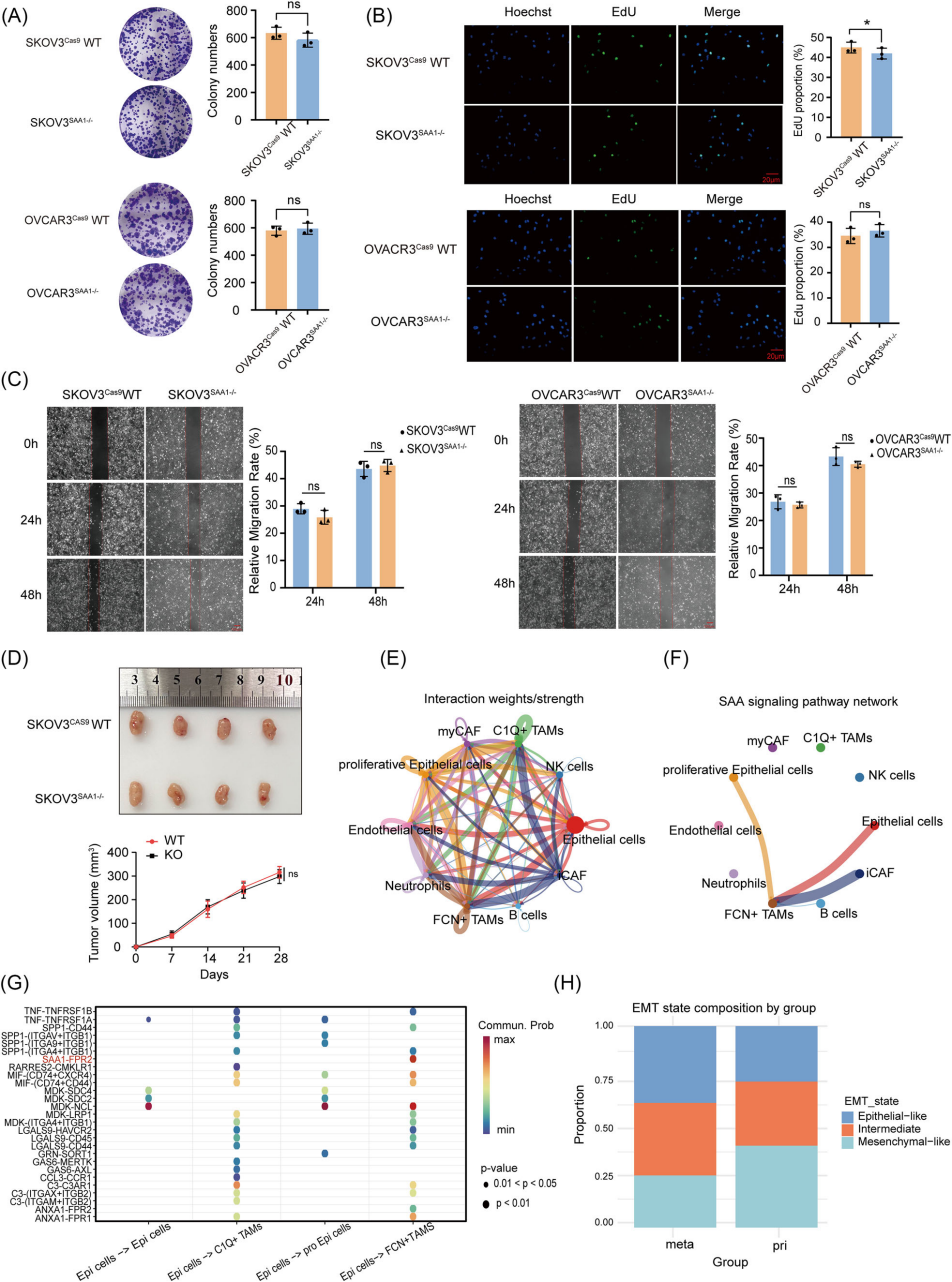
Tumour‐derived SAA1 does not directly promote tumour cell proliferation or migration but drives epithelial–macrophage crosstalk. (A) Colony formation assay after 14 days of culture comparing SKOV3^Cas9^WT vs. SKOV3^SAA1–/–^ and OVCAR3^Cas9^WT versus OVCAR3^SAA1–/–^. (B) EdU staining of WT and SAA1‐KO ovarian cancer cells (SKOV3 and OVCAR3). Representative images showing Hoechst‐labelled nuclei (blue) and EdU‐positive proliferating cells (green). Scale bar, 20 µm. (C) Wound‐healing assays of WT and SAA1‐KO ovarian cancer cells (SKOV3 and OVCAR3). Representative images at 0, 24 and 48 h with quantification of relative migration rate. Scale bar, 100 µm. (D) Subcutaneous tumour growth of WT and SAA1‐KO SKOV3 cells in BALB/c nude mice. Representative tumours and growth curves showing no significant difference (ns). Data represent mean ± SD (*n* = 4). (E) Overall cell–cell interaction network showing communication strength among major cell types in primary ovarian tumours. (F) CellChat analysis of SAA pathway activity in primary HGSOC. (G) Ligand–receptor interactions of SAA1 signalling between epithelial cells and macrophage subsets. (H) Comparison of EMT state composition between primary (pri) and metastatic (meta) tumours. Quantitative data are shown as mean ± SD. Statistical significance in A–C was assessed using unpaired two‐tailed Student’s *t*‐test for the indicated comparisons; tumor growth curves in D were analyzed by two‐way ANOVA. ns, not significant; *p < 0.05; **p < 0.01; ***p < 0.001; ****p < 0.0001.

Given the potential involvement of SAA1 in ovarian cancer progression, we generated SAA1‐knockout SKOV3 and OVCAR3 cell lines using the CRISPR/Cas9 system. Western blot analysis confirmed the complete loss of SAA1 protein expression in knockout clones, while Sanger sequencing revealed frameshift mutations in the coding region (Figure S2A–D). Interestingly, depletion of SAA1 did not markedly affect tumour cell proliferation, colony formation or intrinsic migration ability, indicating that the functional impact of SAA1 on tumour cells likely depends on the tumour microenvironment (Figures 2A–D and S2E–G).

To gain further insight into how the tumour microenvironment regulates SAA1 function, we performed cell–cell communication analysis on tumour specimens. Considering that metastatic initiation occurs at the primary tumour site, our analysis focused on the microenvironment within the primary lesions. Epithelial and proliferative epithelial cells exhibited strong communication with FCN1^+^ TAMs (Figure 2E). Construction of a cell–cell communication network centered on FCN1^+^ TAMs revealed extensive interactions with various cell types, particularly epithelial and proliferative epithelial cells (Figure 2F). Notably, the SAA1–FPR2 ligand–receptor pair was highly enriched in this network. SAA1 expression was primarily detected in epithelial and proliferative epithelial cells, whereas FPR2 was selectively expressed in FCN1^+^ TAMs (Figure 2G). Given that SAA1 is a secreted cytokine, these findings suggest that epithelial cells may secrete SAA1 to activate FPR2^+^ TAMs, thereby mediating tumour–immune interactions within the metastatic microenvironment.

**FIGURE 3:**
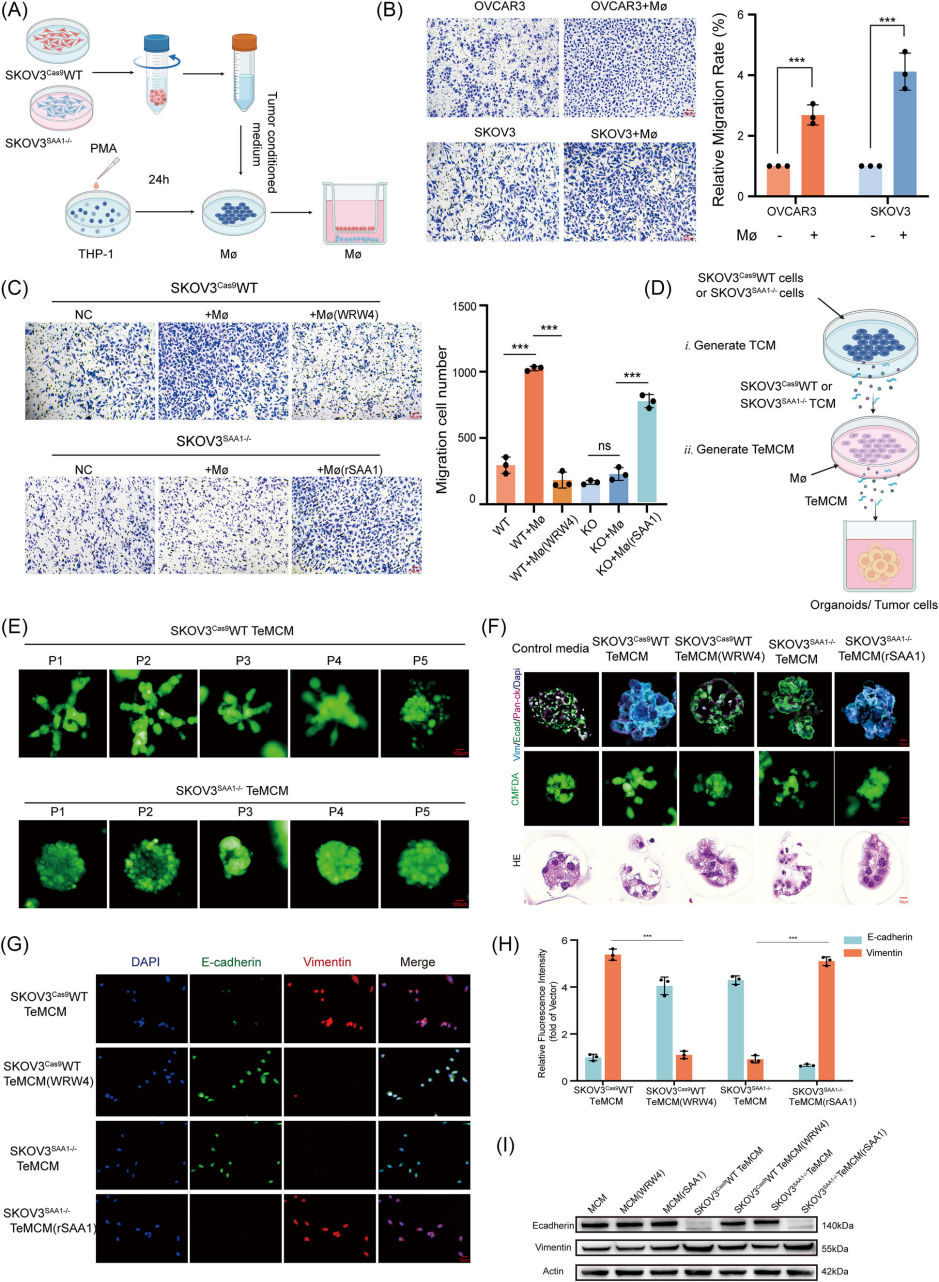
Tumour‐derived SAA1 promotes macrophage‐mediated tumour cell migration and EMT through the SAA1–FPR2 axis. (A) Schematic of the indirect Transwell co‐culture system, with ovarian cancer cells (SKOV3 or OVCAR3; WT or SAA1–/–) seeded in the upper inserts and macrophages (Mø) in the lower chambers. (B) Transwell migration of OVCAR3 and SKOV3 cells with or without macrophages, with representative images and quantification of relative migration rates, *n* = 3. Scale bar, 50 µm. (C) Migration assays of SKOV3^Cas9^WT and SKOV3^SAA1–/–^ cells co‐cultured with macrophages under different conditions, *n* = 3. Representative images (left) and quantification (right). Scale bar, 50 µm. (D) Schematic of a three‐stage cascade co‐culture model: (i) tumour‐conditioned medium (TCM) from WT or SAA1–/– SKOV3 cells; (ii) macrophages exposed to TCM for 24 h to generate tumour‐educated macrophage conditioned medium (TeMCM); (iii) TeMCM applied to ovarian cancer organoids/tumour cells. (E) CMFDA‐stained ovarian cancer organoids cultured with TeMCM from WT or KO SKOV3 cells, *n* = 3. Scale bar, 100 µm. (F) Ovarian cancer organoids cultured under indicated conditions, stained for E‐cadherin (green), vimentin (cyan), pan‐CK (magenta), and nuclei (blue). CMFDA and H&E staining show viability and morphology. Scale bar, 100 µm. (G) Immunofluorescence of SKOV3 cells treated with TeMCM, stained for nuclei (blue), E‐cadherin (green), and vimentin (red). Scale bar, 10 µm. (H) Quantification of fluorescence intensity in G. (I) Western blot analysis of E‐cadherin and vimentin in SKOV3 cells under different MCM or TeMCM treatments. Quantitative data in B, C and H are shown as mean ± SD (*n* = 3 independent experiments). Statistical significance was determined by Student's *t*‐test in B and by one‐way ANOVA with multiple comparisons test in C and H. ns, not significant; *p < 0.05; **p < 0.01; ***p < 0.001; ****p < 0.0001.

We next examined how these tumour–immune interactions affect cellular phenotypes. Analysis of EMT distribution revealed that primary lesions contained a higher proportion of mesenchymal‐like cells, while epithelial‐like cells predominated in metastatic lesions (Figures 2H and S2H). This observation implies that epithelial secretion of SAA1 and subsequent activation of FPR2^+^ TAMs may contribute to EMT dynamics during metastasis. Based on this hypothesis, a tumour–macrophage co‐culture system was established (Figure 3A). The addition of macrophages markedly enhanced tumour cell migration (Figure 3B). Using the FPR2‐specific inhibitor WRW4, which binds directly to FPR2 as verified by thermal shift assays (Figure S3A), we found that macrophages pretreated with WRW4 failed to promote tumour cell migration. Similarly, SAA1‐knockout tumour cells exhibited reduced migratory capacity in co‐culture, while supplementation with recombinant SAA1 restored migration. These findings indicate that the SAA1–FPR2 axis is a key signalling pathway mediating macrophage‐induced tumour cell migration (Figure 3C).

To determine whether tumour‐derived SAA1 promotes malignant phenotypes through macrophage modulation, we established a conditioned medium co‐culture system (Figure 3D). Macrophages were treated with conditioned media derived from SKOV3^Cas9^WT or SKOV3^SAA1–/–^ cells to generate tumour‐educated macrophages (TeMs). Their secreted products (TeMCM) were subsequently applied to patient‐derived organoids (PDOs) or epithelial cells to assess the indirect effects of SAA1‐dependent macrophage activation. These PDOs retained key histological features of their matched parental tumours and expressed ovarian cancer‐associated markers, including p53, Pan‐CK, PAX8 and WT1 (Figure S3B). Organoids exposed to TeMCM from SKOV3^Cas9^WT‐educated macrophages developed invasive morphologies with irregular protrusions, whereas those treated with TeMCM from SKOV3^SAA1–/–^‐educated macrophages remained round and smooth‐edged. Pretreatment of macrophages with WRW4 significantly attenuated the invasive morphology, while supplementation with recombinant SAA1 partially restored it (Figure 3E). The morphological alterations observed in ovarian cancer organoids prompted us to examine whether these changes were associated with EMT activation. Immunofluorescence staining revealed that organoids treated with WT‐derived TeMCM exhibited a typical EMT phenotype, characterized by reduced E‐cadherin and elevated vimentin expression (Figure 3F). In contrast, WRW4 pretreatment or SAA1 knockout blocked EMT induction, whereas recombinant SAA1 supplementation reinstated it (Figure 3G,H). Western blot analysis further confirmed that WT‐derived TeMCM induced a robust epithelial‐to‐mesenchymal transition at the protein level (Figure 3I). Collectively, although SAA1 does not directly regulate tumour cell proliferation or migration, macrophages educated by tumour‐derived SAA1 promote EMT, thereby enhancing the metastatic and invasive potential of ovarian cancer cells. This process is critically dependent on activation of the SAA1–FPR2 signalling pathway.

### 3.3 SAA1–FPR2 signalling promotes macrophage recruitment and polarization

**FIGURE 4:**
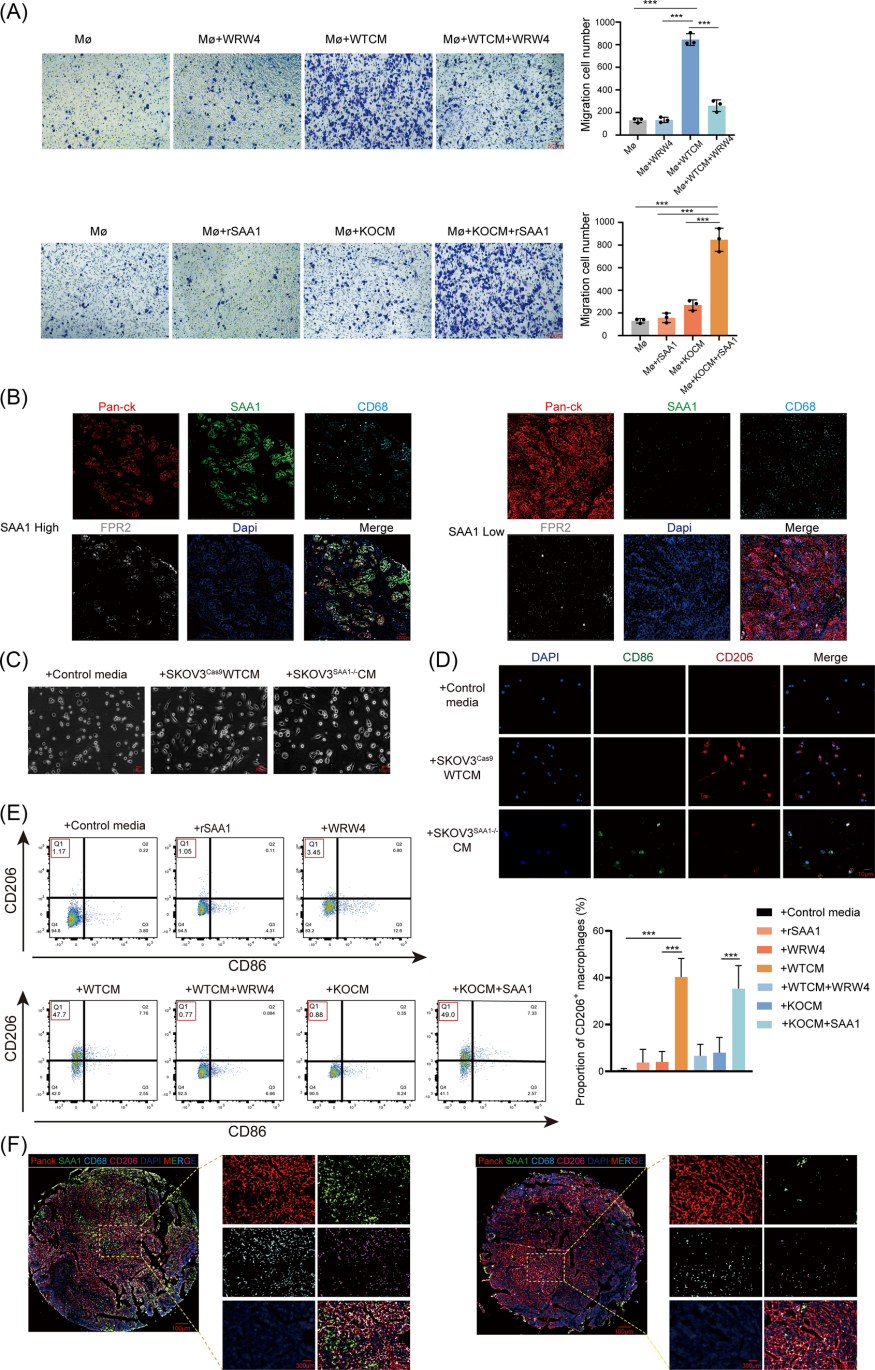
SAA1–FPR2 axis drives macrophage recruitment and polarization. (A) Macrophage migration under WTCM (SKOV3^Cas9^WT CM), KOCM (SKOV3^SAA1–/–^ CM), recombinant SAA1, and/or FPR2 inhibitor WRW4. Representative images (left) and quantification (right). Scale bar, 50 µm. (B) Representative tumour sections from two patients stained for Pan‐CK (red), SAA1 (green), CD68 (cyan), FPR2 (grey), and DAPI (blue), with merged images shown. Scale bar, 20 µm. (C) Representative images showing morphological changes in THP‐1–derived macrophages under the indicated culture conditions. Scale bar, 100 µm. (D) Immunofluorescence staining of THP‐1–derived macrophages under the indicated culture conditions. CD86 (green), CD206 (red), and nuclei (DAPI, blue). Scale bar, 10 µm. (E) Representative flow cytometry plots and quantification of CD206^+^ macrophages under different treatments, including control medium, recombinant SAA1, FPR2 antagonist WRW4, and conditioned media from SKOV3^Cas9^WT (WTCM) or SKOV3^SAA1–/–^ (KOCM), n = 3. (F) Multiplex immunofluorescence staining of SAA1 (green), CD68 (cyan), CD206 (red) and DAPI (blue) in ovarian cancer tissues. Scale bars, 300 µm. Quantitative data in A, D and E are shown as mean ± SD (*n* = 3 independent experiments). Statistical significance was determined by one‐way ANOVA. ns, not significant; *p < 0.05; **p < 0.01; ***p < 0.001; ****p < 0.0001.

Although macrophages were identified as the key cell type mediating the pro‐metastatic effect of SAA1, how SAA1 regulates macrophage function remains unclear. Given the importance of macrophage migration and activation in the tumour microenvironment, we examined whether SAA1 modulates these processes. Our results showed that macrophages displayed strong chemotaxis toward wild‐type tumour cell‐conditioned medium (WTCM), whereas this response was markedly reduced toward SAA1‐knockout medium (KOCM). The FPR2 inhibitor WRW4 blocked WTCM‐induced chemotaxis, while recombinant SAA1 supplementation partially restored it (Figure 4A). In ovarian cancer tissues, tumours with high SAA1 expression exhibited dense CD68^+^ TAM infiltration, whereas SAA1‐low tumours contained sparse macrophages (Figure 4B). Morphologically, macrophages treated with WTCM adopted spindle‐ or stellate‐like shapes characteristic of alternatively activated cells, while those treated with KOCM remained round with smooth edges (Figure 4C). Immunofluorescence analysis showed enhanced CD206 and reduced CD86 expression on macrophages exposed to SKOV3^Cas9^WT conditioned medium, whereas CD206 was markedly reduced in the SKOV3^SAA1–/–^ group (Figure 4D). Consistently, WRW4 treatment significantly reduced the proportion of WTCM‐induced CD206^+^ macrophages, while recombinant SAA1 supplementation restored their levels (Figure 4E). WTCM‐treated macrophages also showed increased expression of immunoregulatory genes, including IL10, TGFB and ARG1, which were modulated by FPR2 inhibition and recombinant SAA1 supplementation (Figure S4A). In patients with ovarian cancer, SAA1‐high tumours contained dense macrophage infiltrates dominated by immunosuppressive CD206^+^ TAMs (Figure 4F). Additionally, in matched serum and tumour tissue samples, serum SAA1 levels were positively associated with both tissue SAA1 expression and CD163^+^ TAM infiltration, suggesting that circulating SAA1 may partially reflect an SAA1‐high, TAM‐enriched tumour microenvironment (Figure S4B). Overall, these findings indicate that SAA1 promotes macrophage recruitment and drives an FPR2‐dependent macrophage reprogramming state with immunoregulatory features, thereby shaping a tumour microenvironment favourable for ovarian cancer progression.

### 3.4 SAA1–FPR2 axis induces CXCL1 secretion from macrophages via JAK2/STAT3 signalling

**FIGURE 5:**
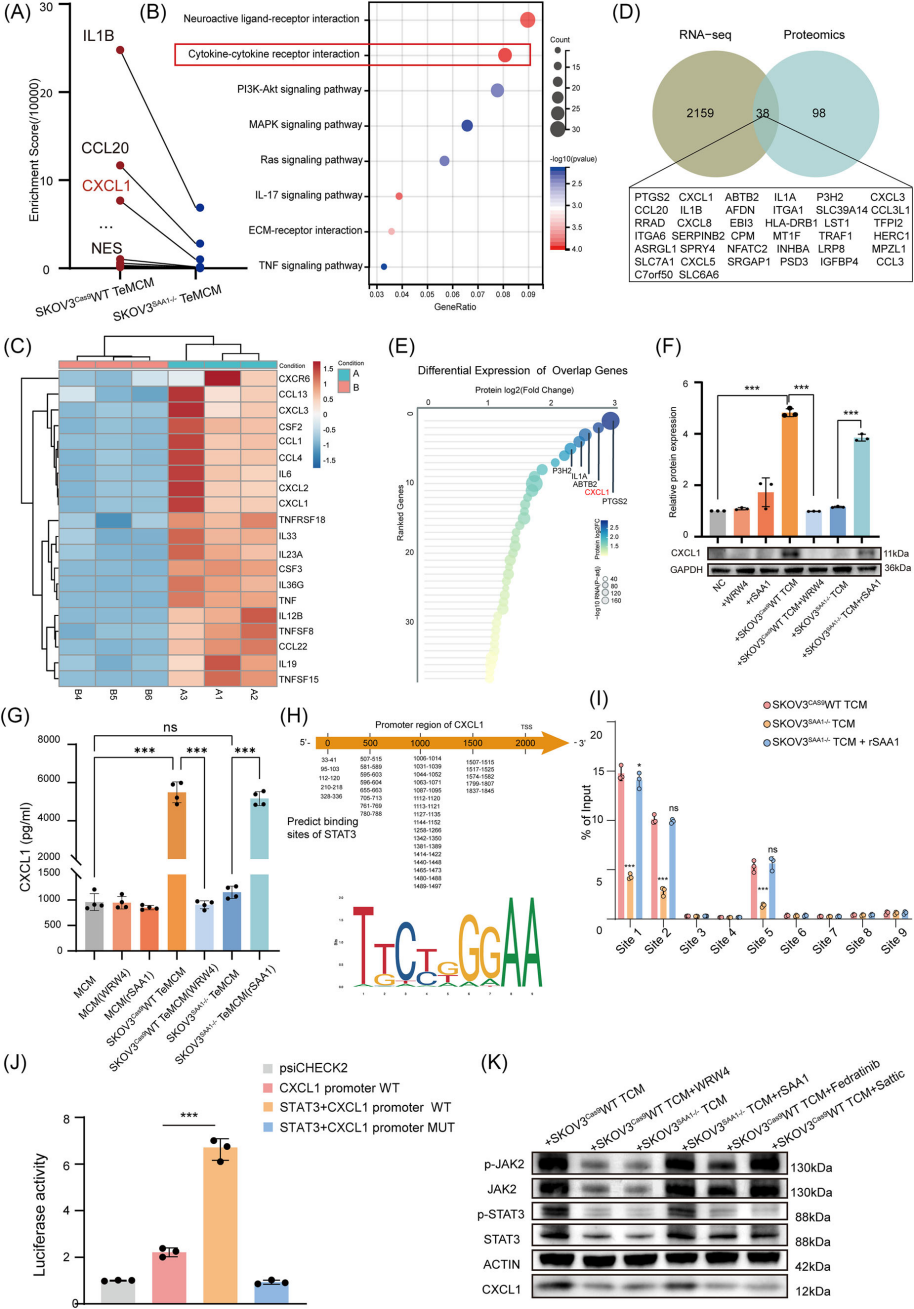
SAA1–FPR2 axis drives CXCL1 production in macrophages via JAK2–STAT3 signalling. (A) Proteomic profiling of secreted factors in TeMCM generated with TCM from SKOV3^Cas9^WT versus SKOV3^SAA1–/–^ cells. (B) KEGG pathway enrichment (bubble plot) of DEGs from macrophages exposed to SKOV3^Cas9^WT versus SKOV3^SAA1–/–^ TCM. (C) Heatmap of cytokine/chemokine‐related DEGs from RNA‐seq of macrophages educated with SKOV3^Cas9^WT TCM (A1–A3) or SKOV3^SAA1–/–^ TCM (B4–B6). (D) Venn diagram showing overlap of upregulated genes from RNA‐seq and proteomics. The 38 shared genes are listed below. (E) Differential expression of the 38 overlapping genes. (F) CXCL1 expression in macrophages under different treatments. Upper: densitometric quantification; lower: representative immunoblots. (G) ELISA quantification of CXCL1 in macrophage supernatants under indicated treatments, *n* = 3. (H) Predicted STAT3‐binding sites within the CXCL1 promoter (−2000 bp to TSS) are shown, along with the consensus STAT3 motif. (I) ChIP–qPCR showing STAT3 enrichment at nine predicted binding sites on the CXCL1 promoter in macrophages treated with SKOV3^Cas9^WT TCM, SKOV3^SAA1–/–^ TCM, or SKOV3^SAA1–/–^ TCM + rSAA1. (J) Dual‐luciferase assay of CXCL1 promoter activity upon STAT3 overexpression, *n* = 3. Reporter groups: empty vector, CXCL1 WT promoter, STAT3 + WT, and STAT3 + MUT. (K) Immunoblot of p‐JAK2, JAK2, p‐STAT3, STAT3, and CXCL1 in macrophages treated with SKOV3^Cas9^WT or SKOV3^SAA1–/–^ TCM with or without WRW4, rSAA1, Fedratinib, or Stattic. Quantitative data in F, G, I and K are shown as mean ± SD (*n* = 3 independent experiments). Statistical significance was determined by one‐way ANOVA. ns, not significant; *p < 0.05; **p < 0.01; ***p < 0.001; ****p < 0.0001.

To investigate the potential role of FPR2 signalling in macrophages during tumour metastasis, we established co‐culture systems using SAA1 wild‐type or knockout tumour cells and macrophages. We then performed integrated proteomic and transcriptomic analyses to identify key downstream secretory factors. Proteomic profiling revealed that, compared with TeMCM derived from SKOV3^SAA1–/–^ cells, TeMCM derived from SKOV3^Cas9^WT cells exhibited marked upregulation of multiple inflammatory cytokines, with IL‐1β, CCL20, and CXCL1 being the most prominently increased (Figure 5A). Consistently, RNA sequencing revealed activation of chemotaxis‐ and inflammation‐related pathways in macrophages exposed to WT‐derived TCM, including CXCL1, CXCL2, CXCL3, and IL6 (Figure 5B–C). Integrated transcriptomic and proteomic analysis identified a set of commonly upregulated candidates in WT‐derived TeMCM, including PTGS2, CXCL1, IL1A and CCL20 (Figure 5D–E). Among these candidates, CXCL1 showed prominent cross‐omics upregulation and was selected for subsequent functional and mechanistic analyses.

We next evaluated whether CXCL1 expression is directly regulated by the SAA1–FPR2 axis. Western blot and ELISA analyses revealed that WT tumour‐conditioned medium (TCM) markedly induced CXCL1 expression, whereas this effect was diminished upon FPR2 inhibition with WRW4. In contrast, TCM from SAA1‐deficient cells failed to upregulate CXCL1, but the addition of recombinant SAA1 (rSAA1) partially restored its expression (Figure 5F–G). These data indicate that CXCL1 induction in macrophages depends on SAA1–FPR2 signalling.

Given that NF‐κB has been implicated in the transcriptional regulation of CXCL1,^20^ we further compared NF‐κB and JAK/STAT signalling in macrophages. JAK/STAT signalling was preferentially enriched and positively associated with CXCL1 expression, whereas NF‐κB signalling was not significantly enriched and p65 phosphorylation showed no clear increase after WT TCM stimulation (Figure S5A–D). These results supported a primary focus on JAK/STAT signalling in this model. To dissect the molecular mechanism underlying this regulation, JASPAR analysis predicted multiple STAT3‐binding motifs within the CXCL1 promoter (Figure 5H), suggesting that the JAK2/STAT3 pathway mediates this transcriptional response. We observed that in macrophages treated with WT TCM, STAT3 binding to the predicted CXCL1 promoter sites (Site 1, Site 2 and Site 5) was markedly enhanced, whereas this binding was substantially reduced in macrophages exposed to SAA1‐deficient TCM. Supplementation with recombinant SAA1 restored STAT3 binding at these sites. No significant changes were detected at the other regions (Sites 3, 4 and 6–9), likely due to the absence of STAT3 recognition motifs or restricted chromatin accessibility that limited binding (Figure 5I). Moreover, luciferase reporter assays demonstrated that STAT3 overexpression significantly increased CXCL1 promoter activity in the WT construct, whereas this effect was lost in the mutant lacking predicted STAT3‐binding sites (Figure 5J). Western blot further showed that WT TCM enhanced JAK2/STAT3 signalling and CXCL1 expression, which were attenuated by WRW4, the JAK2 inhibitor fedratinib, or the STAT3 inhibitor Stattic, whereas rSAA1 restored these effects in macrophages treated with KO TCM (Figure 5K). Together, these findings demonstrate that the SAA1–FPR2 axis activates the JAK2/STAT3 pathway in macrophages, leading to transcriptional upregulation and secretion of CXCL1, thereby establishing a feed‐forward signalling circuit that promotes ovarian cancer metastasis.

### 3.5 CXCL1 promotes invasion and metastasis of ovarian cancer cells

**FIGURE 6:**
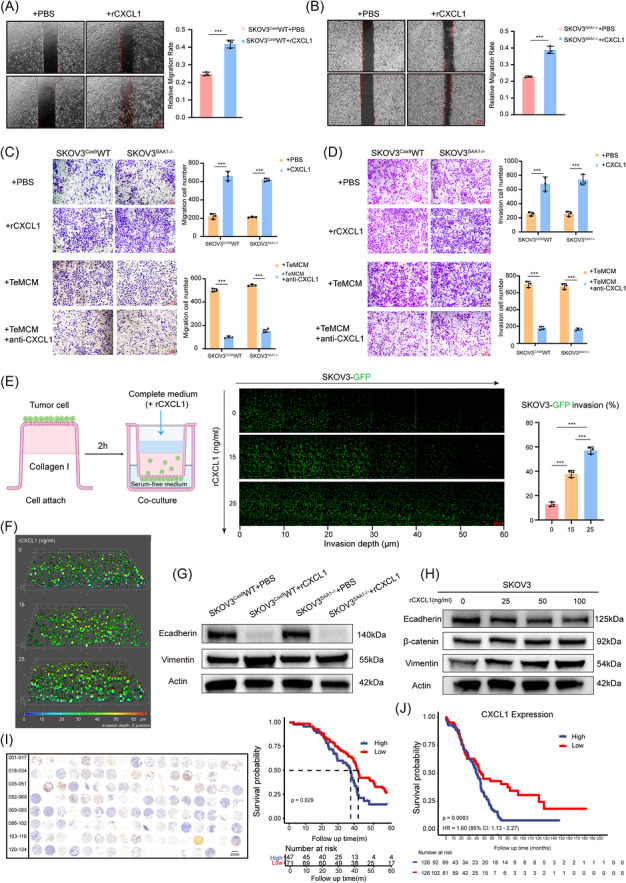
CXCL1 enhances the invasive and metastatic potential of ovarian cancer cells. (A–B) Wound‐healing assays of SKOV3^Cas9^WT and SKOV3^SAA1–/–^ cells treated with rCXCL1 or PBS, *n* = 3. Representative images (left) and quantification of migration rate (right). Scale bar, 100 µm. (C) Transwell migration of SKOV3^Cas9^WT and SKOV3^SAA1–/–^ cells treated with rCXCL1 or PBS, *n* = 3. Representative images (left) and quantification (right). Scale bar, 50 µm. (D) Matrigel invasion assay of SKOV3^Cas9^WT and SKOV3^SAA1–/–^ cells with rCXCL1 or PBS, *n* = 3. Representative images (left) and quantification (right). Scale bar, 50 µm. (E) Left: schematic illustration of the inverse invasion model; middle: laser‐induced fluorescence imaging; right: quantitative analysis of the effect of CXCL1 on SKOV3‐GFP invasion. (F) Representative three‐dimensional reconstruction based on Z‐stack scanning, presented as a depth‐coded heatmap. (G) Immunoblot of E‐cadherin and vimentin in SKOV3^Cas9^WT and SKOV3^SAA1–/–^ cells treated with PBS or rCXCL1. (H) Immunoblot of E‐cadherin, β‐catenin, and vimentin in SKOV3 cells treated with increasing concentrations of rCXCL1 (0–100 ng/mL, 24 h). (I) Representative tissue microarray (TMA) cores stained for CXCL1 by immunohistochemistry. Scale bar, 2 mm. Kaplan–Meier overall survival analysis of the same cohort stratified by CXCL1 IHC score (High vs Low). Log‐rank *p* = 0.029. (J) Kaplan–Meier analysis of TCGA‐OV cohort stratified by CXCL1 mRNA expression (High vs Low). Log‐rank *p* = 0.0083; HR = 1.60, 95% CI 1.13–2.27. Quantitative data in A–E are shown as mean ± SD (*n* = 3 independent experiments). Statistical significance was determined by Student's *t*‐test in A–D and by one‐way ANOVA in E. ns, not significant; *p < 0.05; **p < 0.01; ***p < 0.001; ****p < 0.0001.

To determine whether CXCL1 mediates the pro‐invasive effects of FPR2^+^ macrophages on cancer cells, we performed a series of functional assays. Exogenous supplementation with recombinant CXCL1 significantly enhanced the migratory and invasive capacities of both SAA1 wild‐type and SAA1‐knockout ovarian cancer cells, indicating that the pro‐migratory and pro‐invasive effects of CXCL1 are independent of upstream SAA1 signalling (Figure 6A–D). Consistently, reverse invasion assays demonstrated that recombinant CXCL1 markedly promoted ovarian cancer cell invasion in a dose‐dependent manner (Figures 6E,F and S6A,B), accompanied by increased EMT characteristics (Figure 6G,H), thereby highlighting the role of CXCL1 in driving cancer cell invasion and metastasis. To evaluate the clinical significance of CXCL1, we analysed ovarian cancer tissue microarrays and validated the findings in the TCGA‐OV cohort. High CXCL1 expression was significantly associated with shorter overall survival (Figure 6I,J). Taken together, these data indicate that CXCL1 acts as a key downstream effector of the SAA1–TAM axis, independently driving tumour cell migration and invasion. Moreover, its elevated expression correlates with poor prognosis, underscoring its role as an important promoter of ovarian cancer progression.

### 3.6 Blocking the SAA1–TAM–CXCL1 axis suppresses ovarian cancer metastasis

**FIGURE 7:**
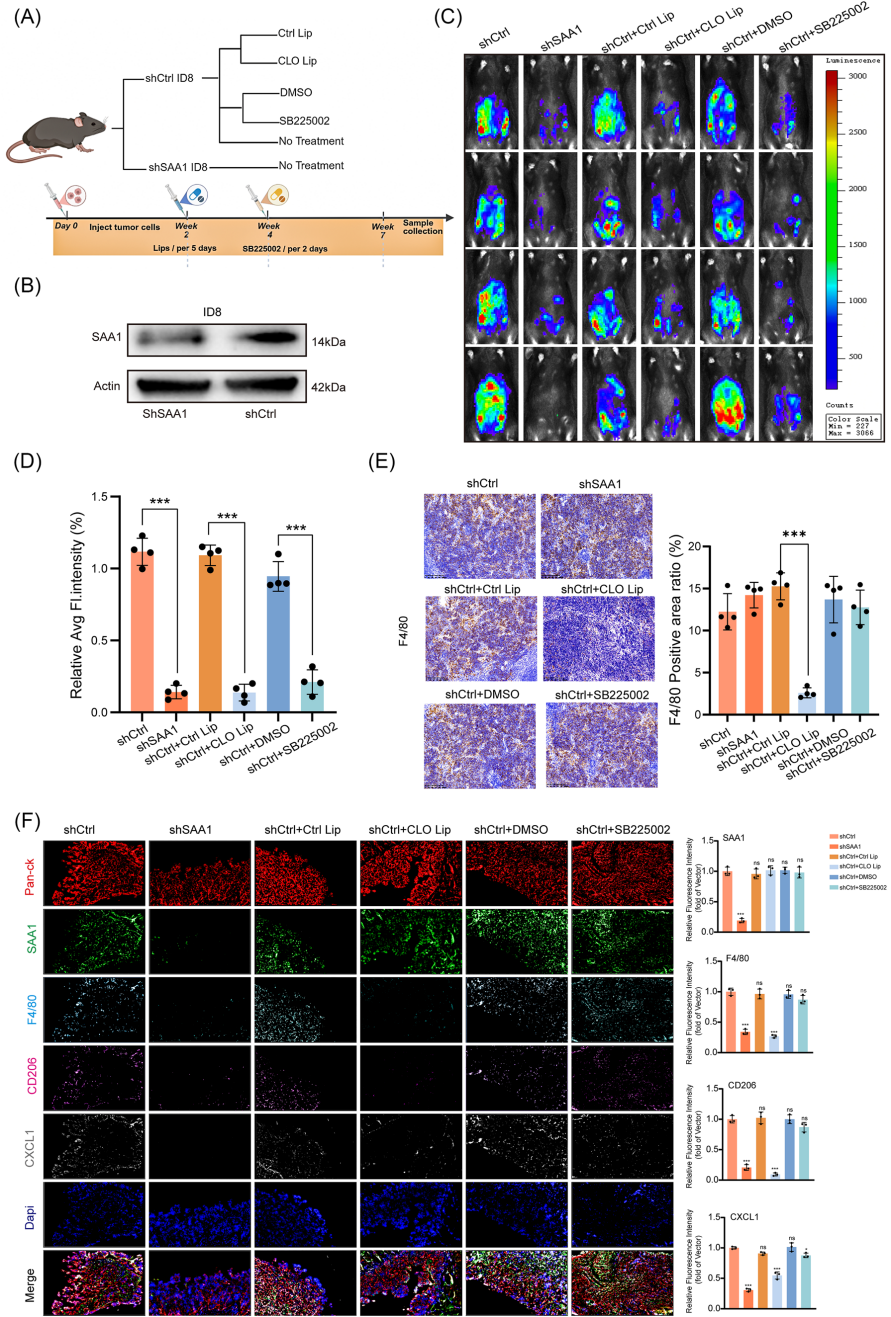
Inhibition of the SAA1–TAM–CXCL1 axis suppresses ovarian cancer metastasis. (A) Experimental scheme for the C57BL/6 mouse model of ovarian cancer peritoneal dissemination. (B) Western blot of SAA1 in luciferase‐labelled ID8 cells. Protein levels were compared between shSAA1‐ID8‐LUC and shCtrl‐ID8‐LUC. (C) Bioluminescence imaging at Week 7 after intraperitoneal injection of ID8‐LUC or shSAA1‐ID8‐LUC cells, treated with control liposomes (Ctrl Lip), clodronate liposomes (CLO Lip), vehicle (DMSO), or the CXCL1 receptor inhibitor SB225002, *n* = 4. (D) Quantification of average luminescence intensity. (E) Immunohistochemistry of F4/80 in spleen sections from mice bearing shCtrl‐ID8‐LUC or shSAA1‐ID8‐LUC tumours, with or without Ctrl Lip, CLO Lip, DMSO, or SB225002. Left: representative fields from red pulp; right: quantification of F4/80^+^ area ratio (%). Scale bar, 100 µm. (F) Multiplex immunofluorescence of peritoneal tumour sections under the indicated interventions. Staining includes Pan‐CK (red), SAA1 (green), F4/80 (cyan), CD206 (magenta), CXCL1 (grey), and nuclei (blue). Merged images are shown in the bottom row. Scale bar, 20 µm. Quantitative data in D–F are shown as mean ± SD. Statistical significance was determined by one‐way ANOVA. ns, not significant; *p < 0.05; **p < 0.01; ***p < 0.001; ****p < 0.0001.

An in vivo ovarian cancer peritoneal dissemination model was established using C57BL/6 mice (Figure 7A). Seven weeks after inoculation, necropsy revealed multiple tumour nodules widely distributed within the peritoneal cavity (Figure S7A). To investigate the role of SAA1, we generated a stable luciferase‐labelled ID8‐LUC ovarian cancer cell line with lentiviral knockdown of SAA1 (shSAA1‐ID8‐LUC) (Figure 7B). Mice were divided into six groups: shCtrl, shSAA1, shCtrl + Ctrl Lip, shCtrl + CLO Lip, shCtrl + DMSO, and shCtrl + SB225002. SAA1 knockdown markedly reduced intraperitoneal tumour burden, while macrophage depletion or CXCR2 inhibition produced similar effects, both significantly suppressing peritoneal dissemination (Figure 7C–D). To determine whether these effects reflected reduced tumour cell proliferation, Ki‐67 IHC was performed on peritoneal tumour tissues. No significant difference in the percentage of Ki‐67^+^ cells was observed among the groups (Figure S7B), including between shCtrl and shSAA1 tumours. These results suggest that the reduced tumour burden was not mainly due to generalized suppression of tumour cell proliferation, but was more closely associated with impaired peritoneal dissemination.

To evaluate biosafety, H&E staining of major organs from each group revealed no evident pathological abnormalities (Figure S7C). In the CLO Lip–treated group, F4/80^+^ macrophages in the splenic red pulp were substantially reduced, confirming effective macrophage depletion (Figure 7E). Multiplex immunofluorescence of peritoneal tumours showed that SAA1 knockdown significantly decreased F4/80^+^ and CD206^+^ TAMs, accompanied by weakened CXCL1 signals, consistent with in vitro observations. These findings indicate that SAA1 loss impairs macrophage recruitment and polarization. Similarly, the CLO Lip–treated group exhibited reduced CXCL1 expression without affecting SAA1 levels, suggesting that CXCL1 is primarily secreted by macrophages rather than tumour cells. In contrast, treatment with the CXCL1 receptor inhibitor SB225002, which targets the downstream step of the tumour‐derived SAA1/TAM axis, effectively blocked CXCL1‐mediated signalling, showing slightly decreased CXCL1 but unchanged SAA1 expression and macrophage distribution (Figure 7F). All the above results suggested that SAA1‐promoted ovarian cancer metastasis depended on the generation of immunosuppressive TAMs via the FPR2–JAK2/STAT3 axis and the ensuing CXCL1 secretion.

## 4 DISCUSSION

In contrast to most solid tumours, ovarian cancer primarily metastasizes through peritoneal dissemination, a process profoundly influenced by tumour–immune interactions.^21^, ^22^, ^23^ Although TAMs are widely recognized for their pro‐tumour roles in metastasis, the molecular basis underlying their reprogramming by tumour cells remains poorly understood.^15^, ^24^, ^25^ Through single‐cell transcriptomic analysis and multilayered experimental validation, this study identifies SAA1 as a key mediator that activates FPR2^+^ TAMs, thereby establishing a signalling pathway that concurrently promotes immunosuppression and metastasis. TAMs in ovarian cancer are highly heterogeneous and can be shaped by their developmental origin, tumour‐derived signals, and local microenvironmental cues within primary tumours, malignant ascites, and peritoneal/omental metastatic niches.^26^ Accordingly, the SAA1‐activated macrophages identified here may represent a tumour‐educated functional state characterized by inflammatory activation, immunoregulatory remodelling, and CXCL1 production, rather than a fixed TAM subset within the traditional M1/M2 framework.

Although numerous studies have emphasized the critical involvement of the TME in metastasis, direct evidence identifying which epithelial subpopulations initiate dissemination remains limited. To address this, we performed single‐cell RNA sequencing on matched primary and peritoneal metastatic lesions from patients with ovarian cancer. The analysis revealed an epithelial subpopulation with high SAA1 expression located at an early developmental branch enriched in migration‐ and inflammation‐related pathways, suggesting its potential role in initiating metastasis. Cell–cell interaction analysis further demonstrated strong ligand–receptor signalling between this subpopulation and FCN1^+^ TAMs, with the SAA1–FPR2 interaction being particularly prominent. These findings imply that SAA1‐high epithelial cells may transmit proinflammatory cues to TAMs at the primary site, contributing to the early formation of a pro‐metastatic microenvironment.

As a classical acute‐phase protein, SAA1 is rapidly upregulated during infection and inflammation, serving as a biomarker of disease activity in conditions such as rheumatoid arthritis and systemic lupus erythematosus.^27^, ^28^, ^29^, ^30^ Beyond inflammation, SAA1 exerts tumour‐promoting and immunomodulatory effects in multiple malignancies. In breast cancer, SAA1 secreted by tumour cells induces immunosuppressive neutrophils through the TLR2/NF‐κB/MAPK pathway, thereby facilitating tumour progression.^31^ In lung adenocarcinoma, SAA attenuates PD‐1–mediated antitumour immunity and promotes fibrosis and Th2 immune polarization.^32^ In non‐malignant inflammatory contexts, SAA1 activates the NOX4/ROS–p38MAPK/NF‐κB pathway in macrophages, leading to proinflammatory cytokine secretion.^33^ Collectively, these studies indicate that SAA1 serves as an immunoregulatory molecule that modulates immune cell activation and inflammatory signalling, thereby influencing both immune responses and tumour progression. Notably, a recent report using lentiviral knockdown and overexpression approaches suggested that SAA1 directly enhances ovarian cancer cell proliferation and metastasis.^34^ However, using CRISPR/Cas9‐mediated complete knockout of SAA1 in SKOV3 and OVCAR3 cells, we found that SAA1 deficiency failed to significantly alter tumour cell proliferation, migration, or growth in xenograft models. This discrepancy may stem from residual expression and off‐target effects associated with lentiviral approaches, which could non‐specifically activate inflammatory signalling and exaggerate the tumour‐promoting effects of SAA1.

Given that SAA1 did not directly enhance the proliferation or migration of ovarian cancer cells, we further examined its tumour‐promoting effects through in vitro co‐culture experiments, hypothesizing that they primarily depend on macrophage‐mediated immune regulation. Previous studies have shown that SAA1 can interact with its receptor FPR2 to activate signalling pathways such as MAPK and NF‐κB, thereby modulating the functional state of the monocyte–macrophage system.^35^, ^36^, ^37^ Our results demonstrated that the SAA1–FPR2 axis strongly promotes macrophage activation and drives their polarization towards an immunosuppressive M2 phenotype. Although SAA–FPR2 signalling has been predominantly reported to promote pro‐inflammatory and M1‐skewed responses in inflammatory diseases, FPR2 has also been shown to mediate immunosuppressive or M2‐like responses in other pathological contexts such as tumours.^36^, ^38^, ^39^ Notably, SAA1‐induced macrophages secreted multiple cytokines that promoted EMT in ovarian cancer cells, significantly enhancing their migratory and invasive capacities. These results indicate that SAA1, by reshaping the immune phenotype of TAMs, establishes a bidirectional amplifying loop between tumour cells and immune cells that facilitates metastasis.

Integrated transcriptomic and proteomic analyses showed that activation of the SAA1–FPR2 pathway profoundly reprogrammed macrophage transcriptional programs, particularly genes related to inflammation and immune regulation, underscoring its role in TAM remodeling. Among the most prominent features of SAA1‐stimulated macrophages was the marked upregulation of CXCL1. Further mechanistic studies showed that this effect depended on sustained activation of the JAK2/STAT3 pathway. FPR2, a canonical G protein–coupled receptor (GPCR), is known to exert dual pro‐ and anti‐inflammatory functions in both inflammatory and tumour signalling contexts.^40^, ^41^ Through Gi protein–dependent mechanisms, it can activate downstream signalling molecules such as MAPK and NF‐κB, playing a critical role in modulating immune responses and the tumour microenvironment.^42^ Although direct evidence for FPR2‐mediated JAK2/STAT3 activation is lacking, STAT3 regulation by other GPCRs suggests potential signalling crosstalk.^43^ Activated STAT3 regulates immune gene expression, including immunosuppressive and proinflammatory chemokines, and promotes M2‐like macrophage polarization.^44^, ^45^ Integrating our data with these prior findings, we propose that SAA1 activates the JAK2/STAT3 pathway via FPR2, inducing robust CXCL1 expression and secretion. This results in a macrophage state characterized by the coexistence of proinflammatory and immunosuppressive features, which not only amplifies macrophage‐mediated tumour promotion but also provides the signalling foundation for CXCL1‐driven tumour–immune cell interactions. The upregulation of CXCL1 thus represents a key molecular mediator of the SAA1–FPR2 axis, delivering a potent downstream signal that fuels tumour cell migration and invasion.

CXCL1 is a proinflammatory chemokine that promotes tumour progression by activating inflammatory paracrine signalling and directly enhancing tumour cell migration and invasion.^46^, ^47^ Acting through its receptor CXCR2, CXCL1 activates ERK, PI3K/AKT, and NF‐κB signalling, leading to EMT induction and increased cellular motility.^48^ In multiple solid tumours, including breast, colorectal, and pancreatic cancers, the CXCL1–CXCR2 axis is widely recognized as a major driver of tumour cell migration, invasion, and metastasis. Its effects involve the NF‐κB/SOX4‐mediated pro‐invasive program observed in breast cancer, as well as downstream CXCR2 signalling pathways that regulate cellular motility.^48^, ^49^, ^50^ In ovarian cancer, the miR‐200 family inhibits CXCL1 (and IL‐8) by direct targeting, thereby blocking pro‐angiogenic pathways such as CXCR2–ERK/MAPK and NF‐κB signalling, which markedly suppresses tumour angiogenesis and distant metastasis.^51^ Our in vitro studies further confirmed this role—exogenous recombinant CXCL1 significantly restored the migratory and invasive abilities of SAA1‐deficient cells, indicating that CXCL1 functions as a downstream effector independently mediating metastasis. In vivo, interventions targeting the SAA1–TAM–CXCL1 axis—including SAA1 knockdown in tumour cells, macrophage depletion, and CXCR2 inhibition—collectively suppressed peritoneal dissemination in mice. These findings highlight the translational potential of this signalling pathway. Importantly, FPR2 serves as a key signalling node linking tumour cells and macrophages within this axis. However, specific pharmacologic inhibitors that selectively target macrophage FPR2 signalling remain unavailable. Thus, functional validation was achieved by manipulating its upstream (SAA1) and downstream (CXCL1/CXCR2) components. Although clinical application of SAA1 inhibitors is currently limited and systemic macrophage depletion may disrupt immune homeostasis, targeting CXCR2 presents a more feasible therapeutic approach. CXCR2 blockade has demonstrated anti‐metastatic effects in several tumour models, and some inhibitors have advanced to clinical trials.^49^, ^52^, ^53^ Taken together, this evidence highlights CXCR2 as a promising therapeutic target for limiting ovarian cancer dissemination.

In conclusion, our study elucidates the pivotal role of the SAA1–TAM–CXCL1 signalling axis in the peritoneal dissemination of ovarian cancer and identifies tumour‐derived SAA1 as the upstream signal driving macrophage functional reprogramming. SAA1 activates the FPR2 receptor on TAMs, triggering the JAK2/STAT3 signalling pathway and inducing CXCL1 secretion, thereby establishing a feed‐forward loop that promotes EMT and metastatic progression of tumour cells. These findings reveal that ovarian cancer dissemination can be achieved through immune remodelling rather than solely relying on intrinsic malignant evolution of tumour cells. This work provides new mechanistic insights into ovarian cancer metastasis and suggests that therapeutic strategies targeting tumour–macrophage interactions may hold significant clinical potential.

## AUTHOR CONTRIBUTIONS

Xuan Zhou and Huangyang Meng developed the methodology, performed data curation and investigation, conducted formal analysis and visualization and drafted the manuscript. Qianjing Chang contributed to methodology development, data collection and manuscript review and editing. Jingjing Ren and Meichen Wen participated in investigation and data collection, and MW provided study resources. Lin Zhang and Wenjun Cheng conceived and supervised the project, administered the study, secured funding and contributed to manuscript review and editing. All authors reviewed and approved the final manuscript.

## CONFLICT OF INTEREST STATEMENT

The authors declare no conflicts of interest.

## ETHICS APPROVAL

All procedures involving animals were approved by the Institutional Animal Care and Use Committee of Nanjing Medical University (Animal Protocol No. IACUC‐2505045). This study was approved by the Ethics Committee of the First Affiliated Hospital of Nanjing Medical University (Approval No. 2023‐SRFA‐218).

## Supporting information



Supporting information

## Data Availability

The raw single‐cell RNA‐seq data generated in this study are available in GSA‐Human under accession number HRA014586 (https://ngdc.cncb.ac.cn/gsa‐human/). The external validation dataset analysed in this study is publicly available in GEO under accession number GSE222556 (https://www.ncbi.nlm.nih.gov/geo/).
