# Supplementary material for: Tumour‐derived SAA1 reprogrammes macrophages to promote CXCL1‐mediated metastasis in Ovarian Cancer
Source: Clin Transl Med. 2026 May 18;16(5):e70698. doi: 10.1002/ctm2.70698 (PMC13181338; doi:10.1002/ctm2.70698)
Supplement: Supplementary file 1 — Supporting information [file CTM2-16-e70698-s001.docx]

**Supplementary Figure legends**


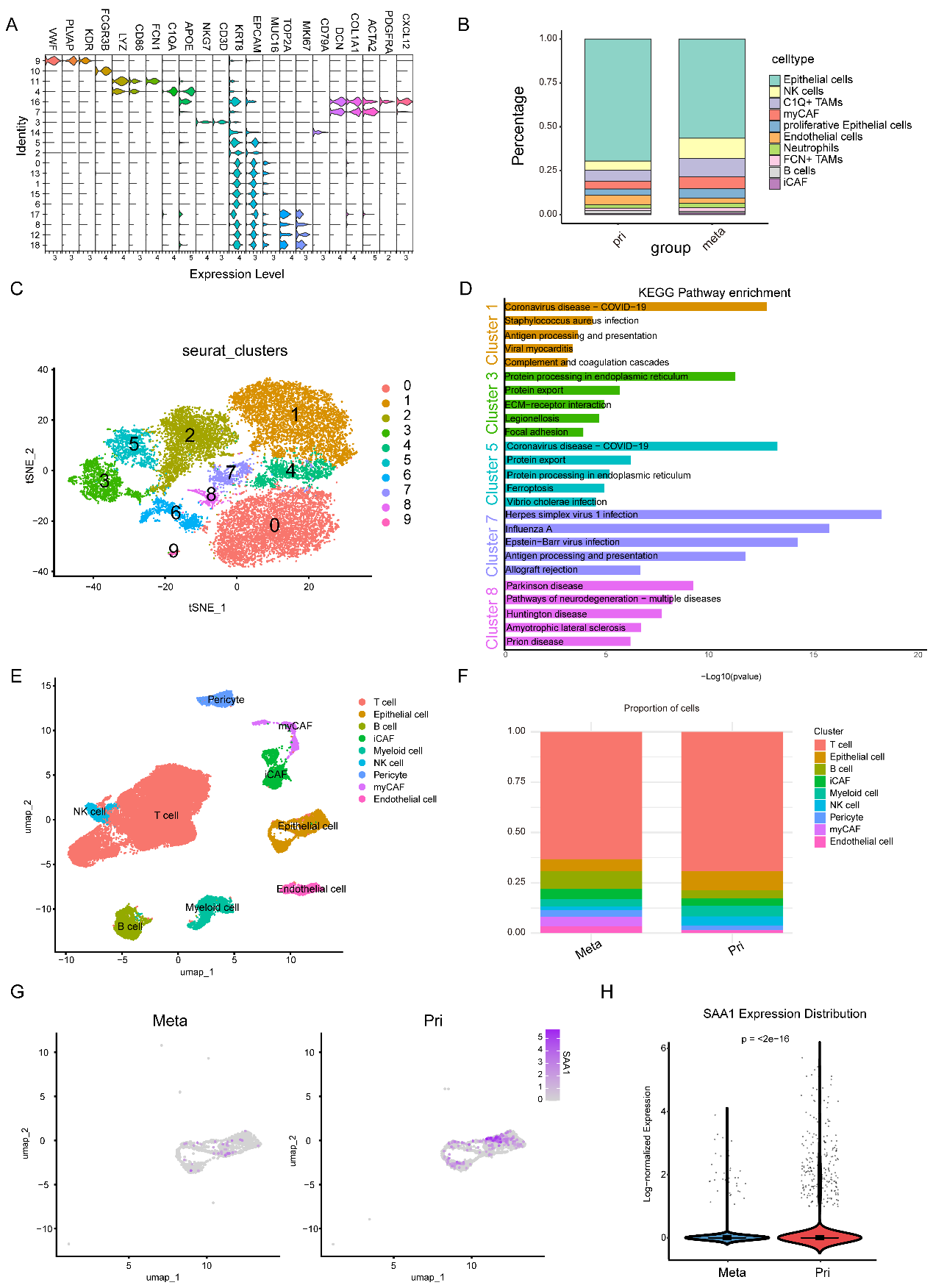


**Supplementary Figure S1.** **Single-cell characterization and validation of SAA1 expression in ovarian cancer.** (A) Violin plots showing representative marker gene expression patterns used to annotate the major cell types. (B) Stacked bar plot comparing the relative proportions of major cell types between primary (Pri) and metastatic (Meta) lesions. (C) Re-clustering of epithelial cells based on highly variable genes, yielding ten distinct clusters (0–9). (D) KEGG enrichment of clusters 1, 3, 5, 7, and 8 based on cluster-specific marker genes. (E) UMAP visualization of major cell populations in the GSE222556 cohort, including untreated ovarian cancer samples with paired primary (Pri) and metastatic (Meta) lesions. (F) Proportions of major cell populations in primary and metastatic lesions. (G) UMAP visualization of SAA1 expression in epithelial cells from primary and metastatic lesions. (H) Violin plots of SAA1 expression in epithelial cells from primary and metastatic lesions. Statistical significance was determined by Wilcoxon rank-sum test.

**
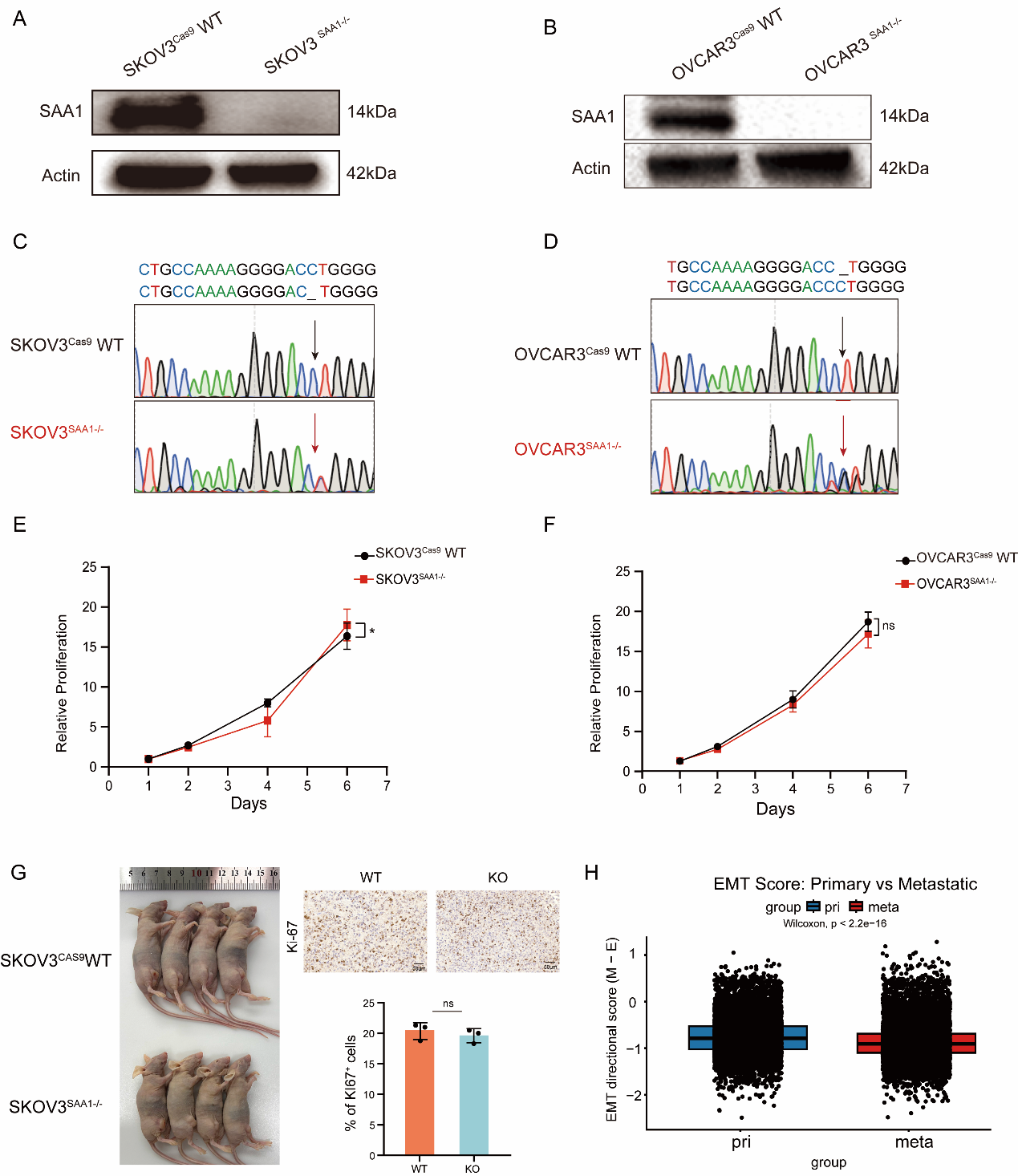
**

**Supplementary Figure S2.** **Assessment of SAA1-knockout tumor cell.** (A-B) Immunoblot of SAA1 in OVCAR3^Cas9^WT and OVCAR3^SAA1-/-^ cells. (C-D) Sanger sequencing chromatograms of SKOV3 and OVCAR3 clones. Representative chromatograms are shown for Cas9 wild-type (Cas9WT) and SAA1 knockout (SAA1–/–) clones. Arrows indicate the indel at the Cas9 cut site in knockout clones. (E-F) CCK-8 assay of cell proliferation in SKOV3^Cas9^WT vs. SKOV3^SAA1-/-^ (left) and OVCAR3^Cas9^WT vs. OVCAR3^SAA1-/-^ (right). (G) Representative BALB/c-nude mice bearing subcutaneous tumors formed by SKOV3^Cas9^WT (top) and SKOV3^SAA1-/-^ (bottom) cells after 4 weeks of inoculation. (H) Comparison of EMT scores between primary (pri) and metastatic (meta) epithelial cells. EMT directional score calculated as mesenchymal minus epithelial signature.

**
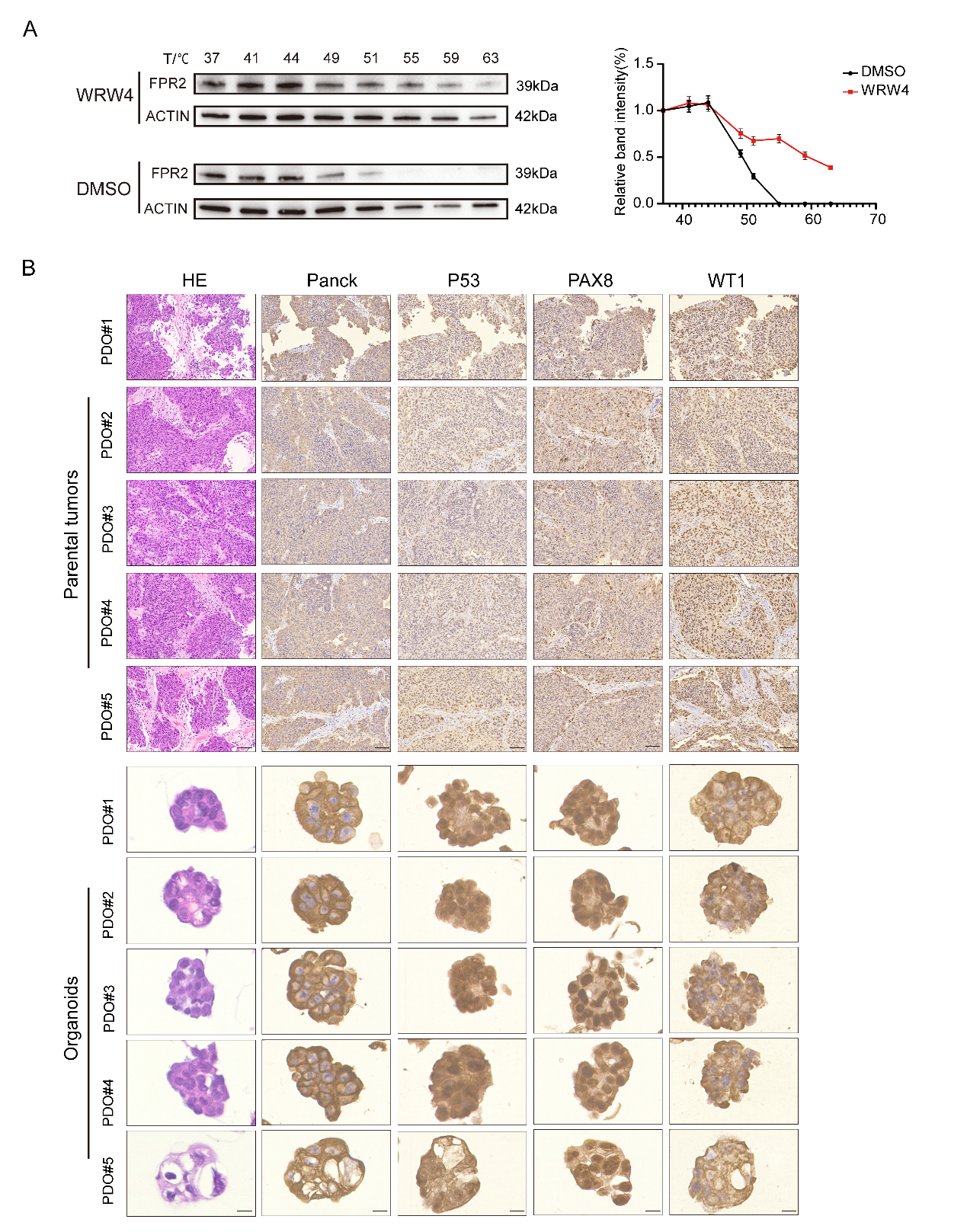
**

**Supplementary Figure S3.** **Validation of FPR2 target engagement and PDO model fidelity.** (A) Immunoblot of soluble FPR2 after heating (37–63 °C) with WRW4 or DMSO treatment, and corresponding normalized melt curves. (B) Representative H&E and immunohistochemical staining for Pan-CK, p53, PAX8, and WT1 in parental tumors and matched PDOs. The PDOs preserved the histological and immunophenotypic features of the corresponding parental tumors. Scale bars, 100 μm for parental tumors and 20 μm for PDOs.


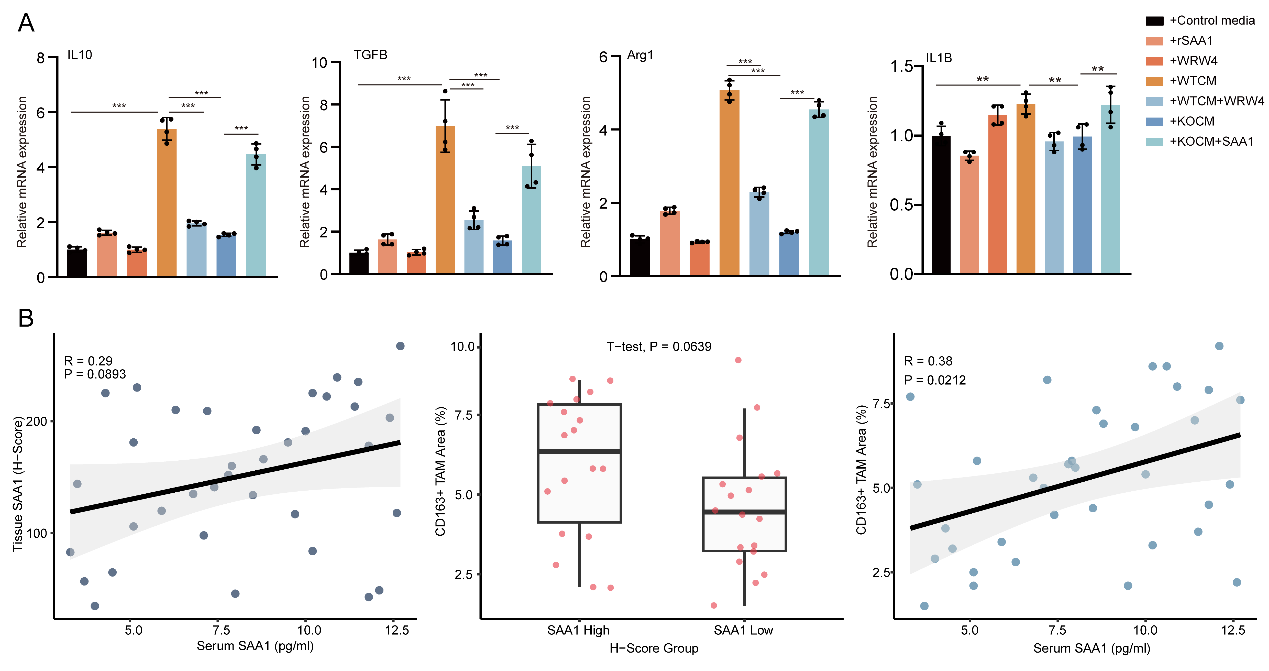


**Supplementary Figure S4. Macrophage remodeling and TAM infiltration associated with tumor-derived SAA1. (**A) qRT-PCR analysis of IL10, TGFB, ARG1, and IL1B mRNA expression in macrophages treated with control medium, recombinant SAA1, WRW4, WTCM, WTCM plus WRW4, KOCM, or KOCM plus recombinant SAA1. Relative mRNA expression was normalized to GAPDH. (B) Relationship between serum SAA1, tissue SAA1 H-score, and CD163⁺ TAM area in HGSOC patients. Tissue SAA1-high/low groups were defined by H-score cutoff: 156. Pearson correlation analysis and unpaired Student’s *t*-test were used. n=36.


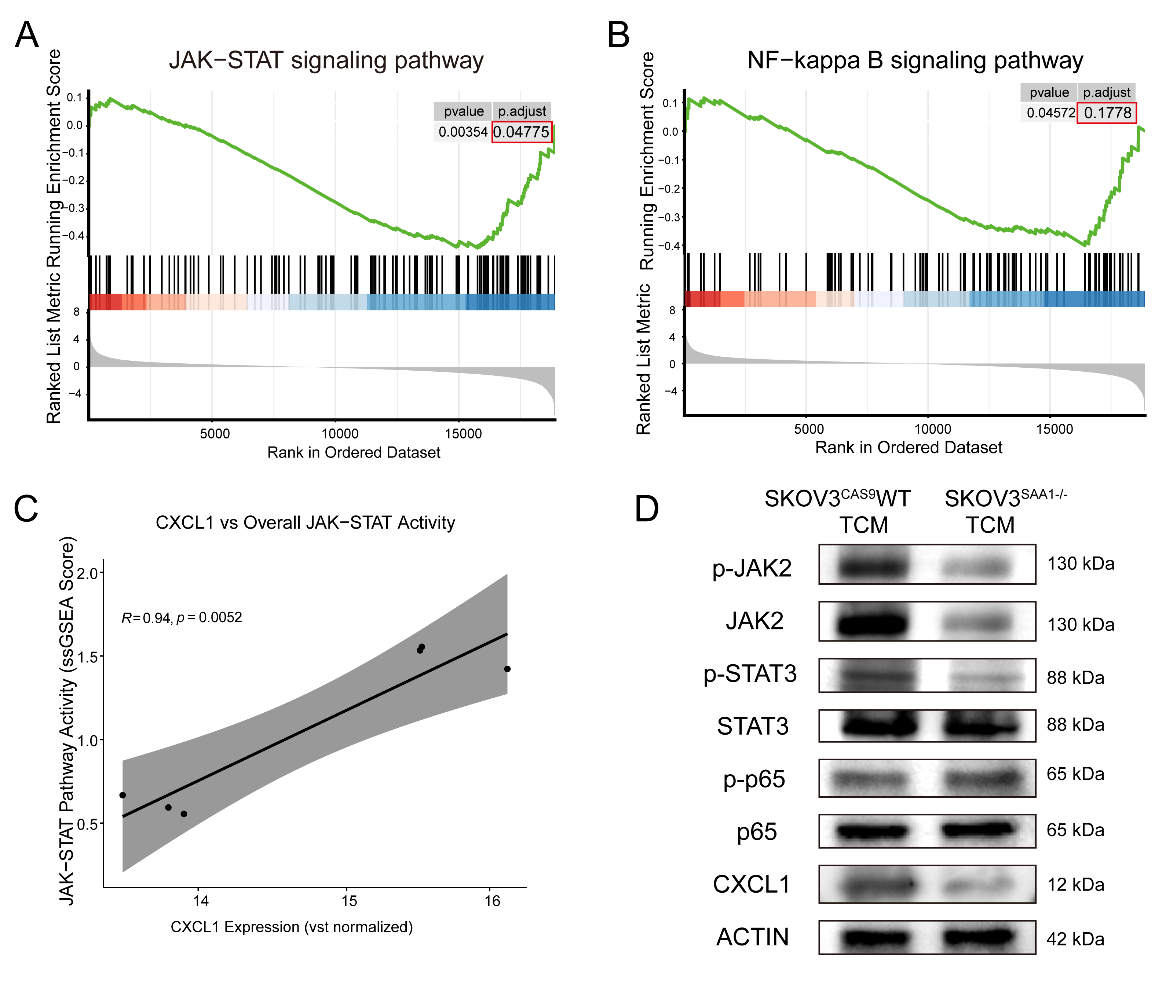


**Supplementary Figure S5.** **Signaling pathway analysis of CXCL1 induction in SAA1-educated macrophages.** (A-B) GSEA plots showing enrichment of the JAK–STAT signaling pathway and NF-κB signaling pathway in macrophages treated with SKOV3^Cas9^WT TCM versus SKOV3^SAA1−/−^ TCM. (C) Correlation between CXCL1 expression and overall JAK–STAT pathway activity. (D) Western blot analysis of p-JAK2, p-STAT3, p-p65, and CXCL1 expression in macrophages treated with the indicated TCM. ACTIN was used as the loading control.


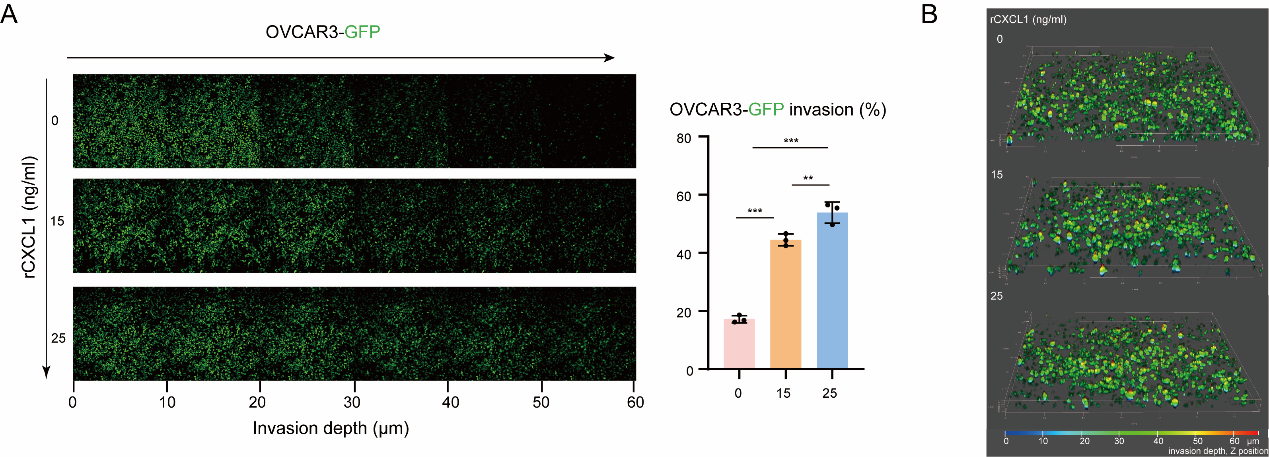


**Supplementary Figure S6.** **CXCL1-induced invasion of ovarian cancer cells.** (A) Left: laser-induced fluorescence imaging; right: quantitative analysis of the effect of CXCL1 on OVCAR3-GFP invasion. (B) Representative three-dimensional reconstruction based on Z-stack scanning, presented as a depth-coded heatmap.


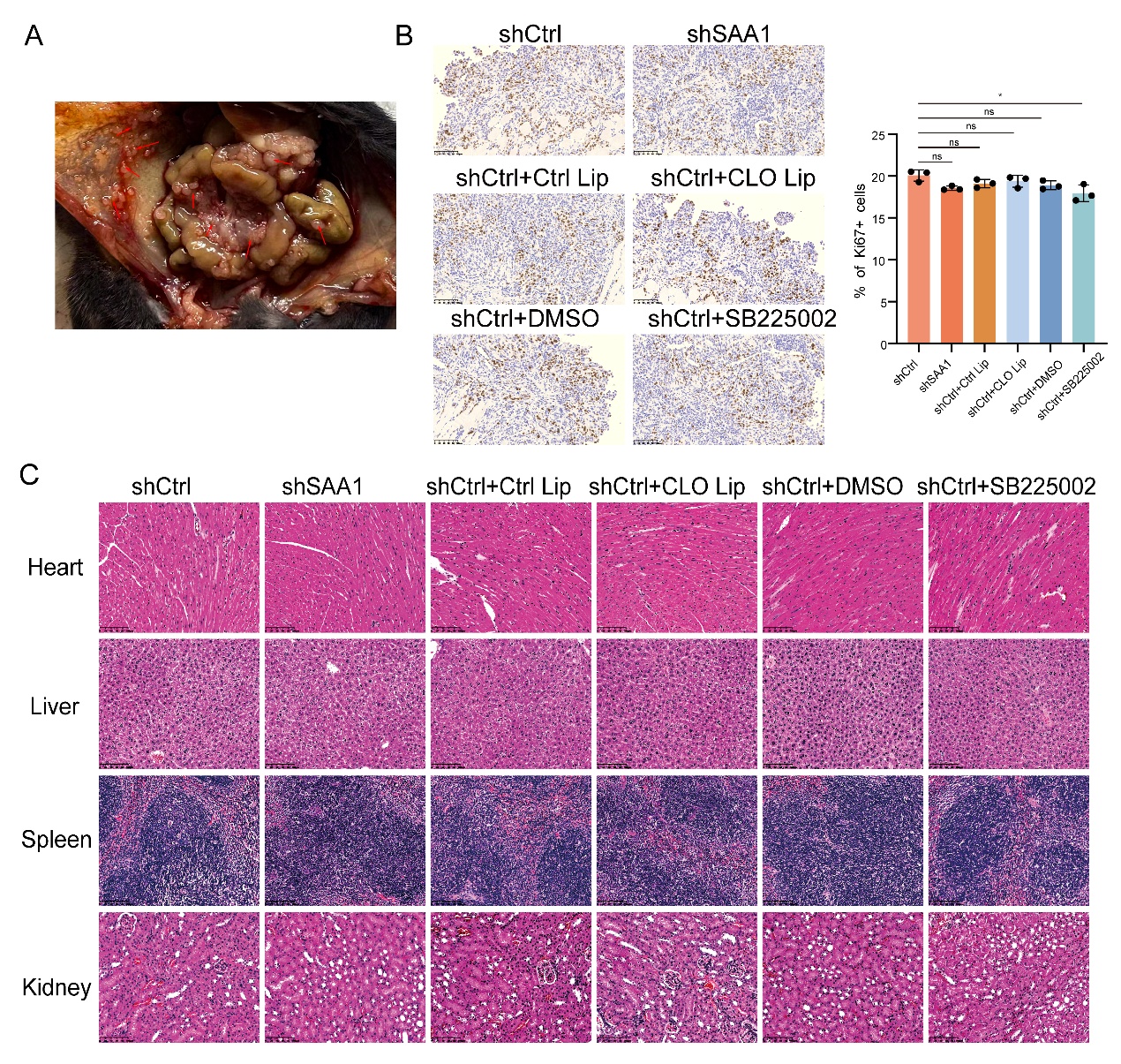


**Supplementary Figure S7.** **In vivo evaluation of peritoneal dissemination, tumor proliferation, and systemic toxicity.** (A) Representative gross image of the peritoneal cavity at 7 weeks after intraperitoneal injection of ID8-LUC cells into C57BL/6 mice, showing multiple disseminated tumor nodules. (B) Representative Ki-67 IHC staining and quantification in tumors from the indicated groups. Scale bar, 100 μm. (C) H&E staining of major organs (heart, liver, spleen, and kidney) from mice in the indicated groups (shCtrl, shSAA1, shCtrl + Ctrl Lip, shCtrl + CLO Lip, shCtrl + DMSO, and shCtrl + SB225002). Scale bar, 100 μm.

**Supplementary** **Table S1.** Antibodies used in this study.

| Company | Antibody | Cat |
| --- | --- | --- |
| Proteintech | Anti-Human CD86 antibody | 65165-1-Ig |
| Proteintech | CD206 Polyclonal antibody | 18704-1-AP |
| Proteintech | E-cadherin Polyclonal antibody | 20874-1-AP |
| Proteintech | Vimentin Monoclonal antibody | 60330-1-Ig |
| Proteintech | Beta Catenin Polyclonal antibody | 51067-2-AP |
| Proteintech | CXCL1 Polyclonal antibody | 12335-1-AP |
| Proteintech | Beta Actin Monoclonal antibody | 66009-1-Ig |
| Proteintech | GAPDH Monoclonal antibody | 60004-1-Ig |
| Proteintech | F4/80 Polyclonal antibody | 29414-1-AP |
| Proteintech | STAT3 Polyclonal antibody | 10253-2-AP |
| ABclonal | SAA1 Rabbit pAb | A14553 |
| ABclonal | FPR2 Rabbit pAb | A23059 |
| ABclonal | JAK2 Rabbit pAb | A7694 |
| ABclonal | Phospho-STAT3-Y705 Rabbit mAb | AP0705 |
| Abcam | Anti-pan Cytokeratin | ab7753 |
| Abcam | Goat Anti-Rabbit IgG H&L (Alexa Fluor® 594) | ab150080 |
| Abcam | Goat Anti-Rabbit IgG H&L (Alexa Fluor® 488) | ab150077 |
| Abcam | Goat Anti-Mouse IgG H&L (Alexa Fluor® 488) | ab150113 |
| Abcam | Goat Anti-Mouse IgG H&L (Alexa Fluor® 594) | ab150116 |
| Biolegend | anti-human CD206 (MMR) | 321109 |
| Biolegend | 7-AAD Viability Staining Solution | 420403 |
| Invitrogen | CD86 (B7-2) Monoclonal Antibody | Invitrogen |

**Supplementary** **Table S2.** Primers for STAT3 binding sites (Site 1–9).

| Site | Forward (5′→3′) | Reverse (5′→3′) |
| --- | --- | --- |
| Site 1 | ATGAGAGCACCAGTACCCCT | TAAATCAAGGCAGGTCCTGG |
| Site 2 | GGCACTTAAATCCATGCTGGC | TAGTAGTGAAGCATCGCCTG |
| Site 3 | TCCTGAGAGAAAACAACATGTGTG | AAATAGGAACGCCGGATCCC |
| Site 4 | TCAGGATGAGTTTCTGTTTAGGCA | CAGCATGGATTTAAGTGCCTTAT |
| Site 5 | GAGTGACAACCAGTGCCGTA | TCCTGCTTATCTTCCTTCTTCCC |
| Site 6 | GTGACAACCAGTGCCGTATTT | TGCTTATCTTCCTTCTTCCCTGT |
| Site 7 | GATCAAACCTGAACCCCTCCT | CGTCAGTGGAAGCCAGGG |
| Site 8 | GCACACCTTTCTACCCTTGACTA | TGTTCATATAAACACCAAAAGCTCA |
| Site 9 | GGACTCGGGATCGATCTGGA | CTCTCCGAGATCCGCGAAC |

**Supplementary Table S3**. Medium recipe (for a total of 50 ml)

| Regents | Work concentration/ 50ml dosage | Manufacturer |
| --- | --- | --- |
| Advanced DMEM/F12 | 43.72 mL | Gibco |
| Glutamax (1x) | 1X | Gibco |
| HEPES (10 mM) | 10 mM | Gibco |
| Pen Strep (%) | 1% | Gibco |
| N-Acetylcysteine (500mM) | 1.25 mM | Sigma-Aldrich |
| Nicotinamide (1M) | 10 mM | Sigma-Aldrich |
| B27 (50x) | 1× | Invitrogen |
| N2 (20x) | 1× | Invitrogen |
| Noggin (100 μg/ml) | 100 ng/ml | Peprotech |
| Rspo1 (250 μg/ml) | 200 ng/ml | Peprotech |
| EGF (500µg/ml) | 5 ng/mL | Peprotech |
| Fgf10 (100µg/ml) | 10 ng/mL | Peprotech |
| A83-01 (5mM) | 0.5 µM | MedChemExpress |
| Y27632 (100mM) | 5 µM | MedChemExpress |
| ITS (%) | 1% | Corning |
| β-Estradiol (100µM) | 100 nM | Sigma-Aldrich |
